# Polymer-induced solid–electrolyte interphase on hard carbon enabling 5C fast-charging practical sodium-ion pouch cell

**DOI:** 10.1093/nsr/nwag025

**Published:** 2026-01-19

**Authors:** Yu Sun, Junjie Du, Tianze Shi, Daxian Zuo, Jiaming Tian, Chengrong Xu, Bo Peng, Jie Yang, Sheng Xu, Yiwen Liu, Yu Shi, Haoshen Zhou, Shaohua Guo

**Affiliations:** College of Engineering and Applied Sciences, Jiangsu Key Laboratory of Artificial Functional Materials, National Laboratory of Solid-State Microstructures, Collaborative Innovation Centre of Advanced Microstructures, Nanjing University, Nanjing 210093, China; Lab of Power and Energy Storage Batteries, Shenzhen Research Institute of Nanjing University, Shenzhen 518000, China; College of Engineering and Applied Sciences, Jiangsu Key Laboratory of Artificial Functional Materials, National Laboratory of Solid-State Microstructures, Collaborative Innovation Centre of Advanced Microstructures, Nanjing University, Nanjing 210093, China; College of Engineering and Applied Sciences, Jiangsu Key Laboratory of Artificial Functional Materials, National Laboratory of Solid-State Microstructures, Collaborative Innovation Centre of Advanced Microstructures, Nanjing University, Nanjing 210093, China; Lab of Power and Energy Storage Batteries, Shenzhen Research Institute of Nanjing University, Shenzhen 518000, China; College of Engineering and Applied Sciences, Jiangsu Key Laboratory of Artificial Functional Materials, National Laboratory of Solid-State Microstructures, Collaborative Innovation Centre of Advanced Microstructures, Nanjing University, Nanjing 210093, China; Lab of Power and Energy Storage Batteries, Shenzhen Research Institute of Nanjing University, Shenzhen 518000, China; College of Engineering and Applied Sciences, Jiangsu Key Laboratory of Artificial Functional Materials, National Laboratory of Solid-State Microstructures, Collaborative Innovation Centre of Advanced Microstructures, Nanjing University, Nanjing 210093, China; Lab of Power and Energy Storage Batteries, Shenzhen Research Institute of Nanjing University, Shenzhen 518000, China; College of Engineering and Applied Sciences, Jiangsu Key Laboratory of Artificial Functional Materials, National Laboratory of Solid-State Microstructures, Collaborative Innovation Centre of Advanced Microstructures, Nanjing University, Nanjing 210093, China; Lab of Power and Energy Storage Batteries, Shenzhen Research Institute of Nanjing University, Shenzhen 518000, China; College of Engineering and Applied Sciences, Jiangsu Key Laboratory of Artificial Functional Materials, National Laboratory of Solid-State Microstructures, Collaborative Innovation Centre of Advanced Microstructures, Nanjing University, Nanjing 210093, China; College of Engineering and Applied Sciences, Jiangsu Key Laboratory of Artificial Functional Materials, National Laboratory of Solid-State Microstructures, Collaborative Innovation Centre of Advanced Microstructures, Nanjing University, Nanjing 210093, China; Lab of Power and Energy Storage Batteries, Shenzhen Research Institute of Nanjing University, Shenzhen 518000, China; College of Engineering and Applied Sciences, Jiangsu Key Laboratory of Artificial Functional Materials, National Laboratory of Solid-State Microstructures, Collaborative Innovation Centre of Advanced Microstructures, Nanjing University, Nanjing 210093, China; Lab of Power and Energy Storage Batteries, Shenzhen Research Institute of Nanjing University, Shenzhen 518000, China; College of Engineering and Applied Sciences, Jiangsu Key Laboratory of Artificial Functional Materials, National Laboratory of Solid-State Microstructures, Collaborative Innovation Centre of Advanced Microstructures, Nanjing University, Nanjing 210093, China; College of Engineering and Applied Sciences, Jiangsu Key Laboratory of Artificial Functional Materials, National Laboratory of Solid-State Microstructures, Collaborative Innovation Centre of Advanced Microstructures, Nanjing University, Nanjing 210093, China; College of Engineering and Applied Sciences, Jiangsu Key Laboratory of Artificial Functional Materials, National Laboratory of Solid-State Microstructures, Collaborative Innovation Centre of Advanced Microstructures, Nanjing University, Nanjing 210093, China; College of Engineering and Applied Sciences, Jiangsu Key Laboratory of Artificial Functional Materials, National Laboratory of Solid-State Microstructures, Collaborative Innovation Centre of Advanced Microstructures, Nanjing University, Nanjing 210093, China; Lab of Power and Energy Storage Batteries, Shenzhen Research Institute of Nanjing University, Shenzhen 518000, China

**Keywords:** fast charging, hard carbon, polymer, solid–electrolyte interphase

## Abstract

Achieving <15 min fast-charging technology for long-life sodium-ion batteries (SIBs) remains a formidable challenge, primarily due to parasitic reactions and unstable solid–electrolyte interphase (SEI) at the hard carbon (HC) interface. Here we develop a universal polymer-induced SEI strategy that enables an Ah-level SIB pouch cell to achieve <10 min fast-charging capability. We design a <4.0 nm functionalized polymer molecular layer, polyethylenesulfonyl fluoride (PESF), coated on the HC surface (PolyHC) to minimize electrolyte decomposition. The PESF with the –SO_2_F group attached has a powerful polar feature, which simultaneously induces an anion enriched at the PolyHC interface and tailors extra F atoms, contributing to the architecture of a ∼5.0 nm stable SEI that hybridizes polymer and NaF. This SEI with a resilient polymer skeleton permanently holds the generated inorganic component, enabling long-term structural stability during fast charging. The assembled 1.2 Ah pouch cell, paired with NaNi_1/3_Fe_1/3_Mn_1/3_O_2_ cathode and PolyHC anode, displays exceptional fast-charging capability and durability. This method is compatible with various HCs, offering a novel perspective for modulating the HC interface chemistry.

## Teaser

A universal polymer-driven hard carbon anode interface regulation strategy empowers an Ah-level sodium-ion pouch cell to achieve 5C fast charging and extended longevity.

## INTRODUCTION

Sodium-ion batteries (SIBs), a key technology for next-generation energy storage systems, are emerging and have received international attention due to their potential low costs and resource advantages [1–3]. To enhance market competitiveness, SIBs are expected to match the fast-charging benchmarks of lithium-ion batteries (the United States Advanced Battery Consortium set a fast-charging time target for LIBs of <15 min) [4,5]. However, achieving both <15 min fast-charging capability and longevity remains a formidable challenge for currently available SIBs [6–8].

The anode side is considered to be the key factor hindering the fast-charging capability of SIBs due to complex parasitic side reactions and slow interface transport dynamics [9,10]. Owing to its intrinsic turbostratic disorder, hard carbon (HC), with characteristically enlarged interlayer spacing (>0.36 nm) and abundant nanoscale porosity, currently stands as the most suitable commercially scalable anode for SIBs [11,12]. The distinctive structural configuration of HC enables >300 mAh g⁻¹ of reversible sodium storage capacity supported by a synergistic ‘intercalation–adsorption–nanopore-filling’ mechanism (Fig. S1) [13,14]. Additionally, the highly isotropic dilation and contraction of carbon interlayers during sodiation/desodiation confer exceptional structural stability to HC, thereby enhancing cycling stability and extending calendar life. Unfortunately, commercially available HCs still suffer from persistently low initial Coulombic efficiency (ICE, mostly <90%) and poor fast-charging capability (charging time >30 min), critically impeding their deployment in practical SIBs [15,16].

The HC–electrolyte interface chemistry, determining solid–electrolyte interphase (SEI) formation, critically governs these performance limitations [17,18]. Three factors strongly connected to SEI formation synergistically restrict the fast-charging capability of HC: (i) weak desolvation capacity at the interface [19,20]—the slow escaping of sodium ions from the solvated structure at the interface (desolvation process) significantly affects the fast-charging behavior of SIBs; (ii) severe parasitic side reactions at the interface [21,22]—the electrolyte decomposes uncontrollably at the HC interface, triggering numerous parasitic side reactions, which severely deplete the electrolyte and thicken the SEI, thereby increasing irreversible sodium loss (low ICE) and interface impedance (poor fast-charging capability); and (iii) a brittle SEI with instability and repetitive reformation [23,24]. During fast sodium ion insertion, the pronounced volume change and local electric field inhomogeneity at the HC surface exacerbate the SEI fracture, exposing fresh carbon surfaces to electrolyte decomposition [23,24]. This results in continuous SEI thickening, increased interfacial impedance and severe polarization, ultimately undermining both fast-charging capability and cycling stability.

The engineering of a stable, homogeneous and low-impedance SEI on commercial HC surfaces remains a significant challenge, yet constitutes a critical imperative for enabling the commercial viability paradigm of SIBs [25,26]. Substantial endeavors have been dedicated to addressing these challenges, with principal strategies encompassing presodiation protocols, passivating coating and advanced electrolyte engineering (Fig. S2) [27–29]. Notwithstanding the efficacy of presodiation protocols in enhancing the ICE of commercial HC anodes, such treatments fail to orchestrate electrolyte-derived formation of a stable SEI while simultaneously exhibiting commercially prohibitive processing costs [27,30]. Additionally, passivating coatings effectuate physical decoupling at the HC–electrolyte interface, thereby mitigating parasitic decomposition reactions and concomitantly elevating ICE through strategic isolation of electroactive surfaces [28]. However, the concomitant processing intricacies and prohibitive economic overheads intrinsic to this technique fundamentally constrain its commercial scalability. Advanced electrolyte engineering induces preferential decomposition of anions at the HC interface to generate an inorganic component-rich SEI, thereby minimizing electrolyte decomposition and constructing a stable SEI. Regrettably, achieving precise orchestration of anion preferential decomposition at HC interfaces within complex electrochemical environments remains a formidable scientific challenge [29,31]. A pressing market-driven imperative exists for effective strategies that concomitantly enhance the ICE of commercial HC and facilitate stable SEI formation—a pivotal enabler for accelerating commercial deployment of SIBs with <15 min fast-charging capabilities [27–29,31,32].

Here we develop a universal polymer-induced SEI strategy that enables an Ah-level SIB pouch cell to achieve 5C fast-charging capability. A <4.0 nm polymer, polyethylenesulfonyl fluoride (PESF), attached by an –SO_2_F group was exquisitely coated *in situ* on the HC surface (PolyHC) by a simple solvent gradient evaporation process. This PESF polymer functionally reshapes the solvation structure of the HC interface, prompting anion enrichment at the interface while repelling the solvent. Additionally, the PESF can synchronously dissociate F atoms that synergistically enriched anions to construct a robust and ∼5.0 nm SEI with polymer and NaF hybridization. PolyHC exhibits superior rate performance in both ester and ether electrolytes, and achieves stable, continuous cycling for 1500 cycles at a high current density of 0.5 A g^–1^ in ester electrolytes. To verify its practical potential, the assembled 1.2 Ah SIB pouch cell, matched with an NaNi_1/3_Fe_1/3_Mn_1/3_O_2_ (NFM) cathode and PolyHC anode, achieved fast-charging capability of <10 min and has excellent cycle stability and durability (capacity retention ∼70% after 1000 cycles, 5C/5C). Expanding this, we verified the feasibility of this polymer coating technology on another five types of commercially available HC.

## RESULTS AND DISCUSSION

### Design concept of polymer-induced SEI strategy

**Figure 1.**
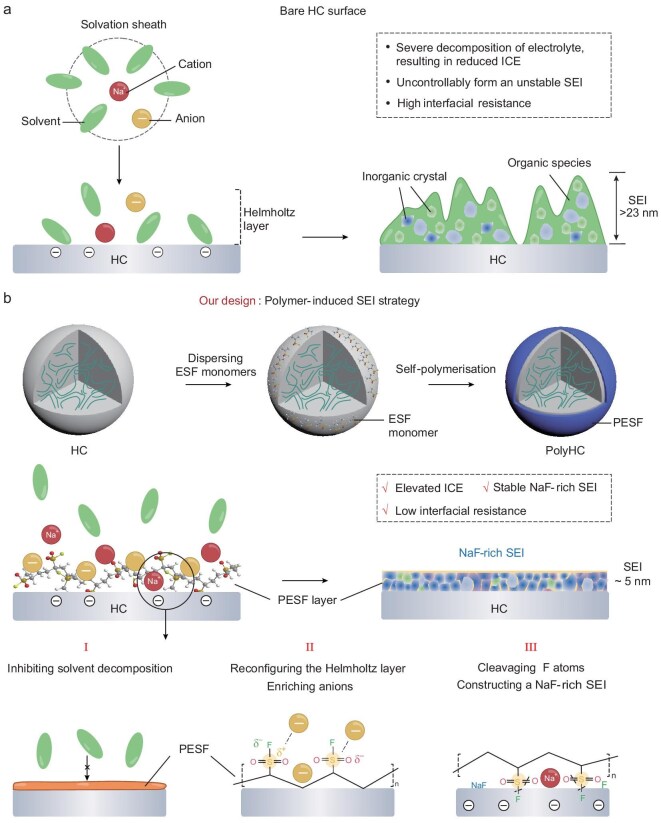
The design concept of the polymer-induced SEI strategy. (a) Bare HC interface chemical analysis and SEI model formed on its surface. (b) Preparation process of the PESF polymer coating of HC and the interface chemical evaluation of the PolyHC surface. PolyHC functionally inhibits electrolyte decomposition, enriches anions, and supplies F atoms, thereby synergistically constructing a robust and thin SEI with polymer and NaF hybridization.

The low ICE and unsatisfactory fast-charging behavior of commercial HC are profoundly correlated with the uncontrollable decomposition of the electrolyte and the formation of SEI at its interface (Fig. 1a and Fig. S1). Excessive decomposition of solvents inevitably triggers an increase in organic components that induces irregularity in the SEI and raises interface impedance. Meanwhile, the organic components of the electrolyte decomposition have been previously confirmed to dissolve into the electrolyte during the process, which may expose fresh electrode interfaces to further deplete the electrolyte and retard the transport kinetics of ions in the electrolyte [33,34]. If a robust SEI can be formed on the HC surface during the initial cycles, undoubtedly this will significantly suppress electrolyte consumption and minimize SEI thickness, thereby enhancing its ICE and fast-charging capability. Considering the above aspects, we fabricated a ∼4.0 nm PESF polymer layer *in situ* on the HC surface (PolyHC) by using a simple solvent gradient evaporation method (Fig. 1b and Figs S3–S7). PolyHC coated with a polymer layer directly prevents contact between the electrolyte and the HC, minimizing electrolyte consumption. Interestingly, PESF is connected to a highly polar –SO_2_F group (Fig. S8), which reconfigures the Helmholtz layer and alters the solvation structure at the PolyHC interface. As a result, PESF is capable of prompting anion enrichment at the interface of PolyHC, which is subsequently verified by molecular dynamics (MD) simulation calculations. At the same time, the –SO_2_F groups within PESF can dissociate F atoms to assist in the formation of a favorable SEI enriched with NaF (Fig. 1b). Consequently, a ∼5.0 nm thin, uniform and robust SEI, featuring a hybrid of NaF and polymer layers, was engineered on the PolyHC interface. As a comparison, the commercial HC surface forms a >25.0 nm-thick non-uniform SEI, which results in substantial irreversible sodium loss (low ICE) and impaired cycling stability, particularly at fast-charging conditions.

### Interfacial chemistry analysis of PolyHC

**Figure 2.**
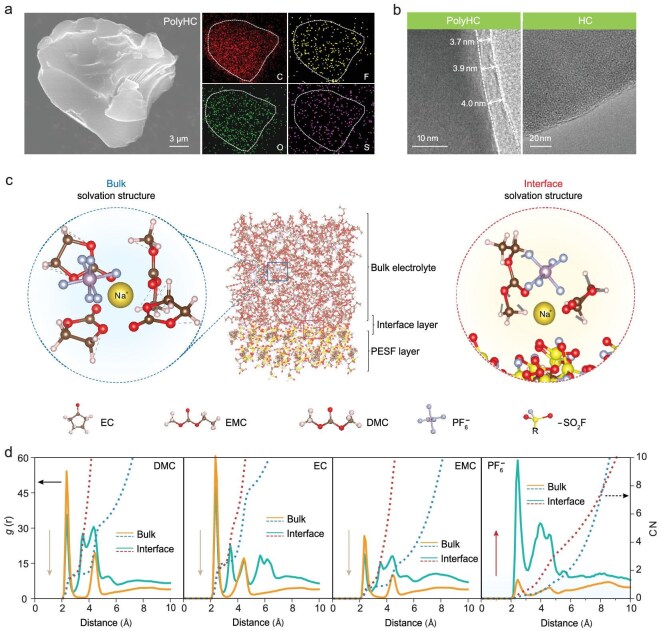
Interfacial chemistry analysis of PolyHC. (a) SEM image of PolyHC and its surface element mapping distribution. (b) TEM images of PolyHC and HC surfaces. (c) MD simulations of the PESF polymer layer within 1 M NaPF_6_ in the EC/EMC/DMC electrolyte system. Contrasting MD snapshots depicting representative solvation configurations in the bulk electrolyte versus at the interfacial region. (d) Quantitative spatial mapping of electrolyte species (solvent molecules and anions) derived from MD simulations reveals distinct distribution profiles between the PolyHC interface and bulk electrolyte domains.

To confirm the successful coating of PESF polymers on HC, we employed scanning electron microscopy (SEM) and transmission electron microscopy (TEM) for detection. The results show that the inherent elements of PESF, including C, O, S and F, are homogeneously distributed on the PolyHC surface, revealing that PESF is properly coated on bare HC (Fig. 2a and Figs S9 and S10). Additionally, Fourier transform infrared (FTIR) spectroscopy also successfully detected the characteristic peak of PESF on the PolyHC surface, further verifying the successful coating of the polymer (Fig. S11). Another consideration is the effect of the polymer layer thickness for ICE. We investigated the impact of different ESF monomer concentrations on the ICE of commercial HC (Fig. S12). Owing to its inherent consumption of sodium ions, an excessively high ESF monomer concentration results in increased irreversible sodium loss. Conversely, lower concentrations lead to incomplete polymerization, thereby compromising the enhancement of ICE. As a result, ESF monomer molecules at a concentration of 0.03 g L^–1^ are demonstrated to effectively elevate the ICE of selected commercial HC to 90% (Fig. S12c). To detect the coating thickness of PESF polymers, we employed TEM to visualize the interface of PolyHC and bare HC (Fig. 2b). A uniform PESF polymer overlayer exhibiting a <4.0 nm thickness is discerned on the PolyHC substrate, in stark contrast to the pristine, uncoated surface morphology observed for bare HC. The encapsulation by the PESF polymer prevents direct electrolyte–electrode interfacial contact, thereby effectively mitigating excessive electrolyte depletion during initial cycling.

Considering the inherently high polarity of PESF (Fig. S8), it may affect the solvation structure of the electrolyte at the PolyHC interface. To probe the interfacial effects of PESF on electrolyte behavior, MD simulations were employed, with computational results presented in Fig. 2c. The –SO_2_F group mediates a distinct reorganization of interfacial solvation configurations, notably divergent from solvation structures in the bulk electrolyte. The spatial distributions of electrolyte constituents, solvent molecules [ethylene carbonate (EC), methyl ethyl carbonate (EMC), dimethyl carbonate (DMC)] and anions (PF_6_⁻), across the PolyHC interface are quantitatively mapped to elucidate the regulatory role of the PESF polymer (Fig. 2d). The PolyHC interface exhibits a substantial reduction in the spatial density distribution of solvent molecules relative to bulk electrolyte. Conversely, a marked interfacial enrichment of anions (PF_6_⁻) occurs at the PolyHC interface, primarily attributable to electrostatic attraction effects induced by the polar PESF polymer. Interfacial anion enrichment facilitates preferential reduction decomposition during initial cycling, generating a robust, NaF-rich SEI.

### Electrochemical performance assessment

**Figure 3.**
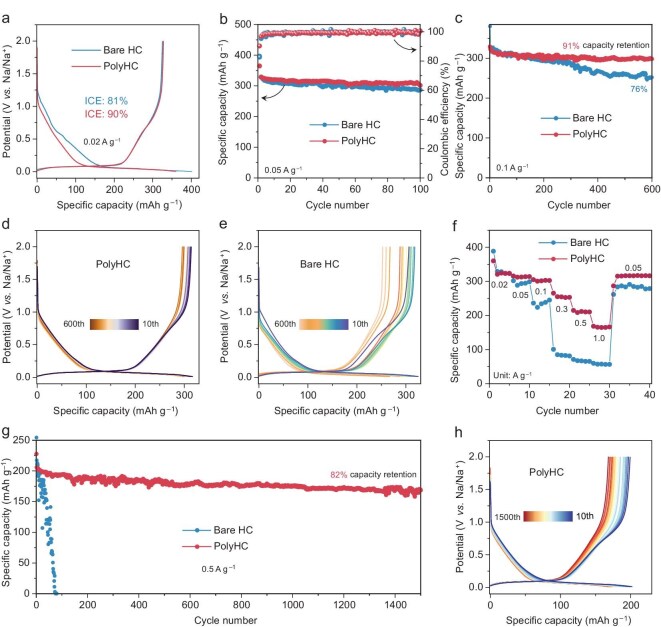
Electrochemical performance evaluation under ester electrolyte (1 M NaPF_6_ in EC/EMC/DMC) conditions. (a) The GCD curves of bare HC and PolyHC at 0.02 A g^–1^. (b) PolyHC and bare HC tested over 100 cycles at 0.05 A g^–1^. (c) The durability performance of PolyHC and bare HC at 0.1 A g^–1^ and (d and e) the corresponding GCD curves. (f) The rate performance of bare HC and PolyHC form 0.02 to 1.0 A g^–1^. (g) The longevity and stability of PolyHC and bare HC evaluated at a high current density of 0.5 A g^–1^ and (h) the corresponding GCD curves of PolyHC.

To evaluate the feasibility of the polymer coating technology, we systematically characterized both bare HC and PolyHC electrodes using a typical ester electrolyte, 1 M NaPF_6_ in EC/EMC/DMC. Figure 3a presents the galvanostatic charge–discharge (GCD) curves of bare HC and PolyHC at a current density of 0.02 A g^–1^. Compared to bare HC (81%), the ICE of the PESF-coated PolyHC increased to 90%. This improvement in ICE can be ascribed to the pronounced attenuation of electrolyte decomposition at the PolyHC/electrolyte interface, as manifested by the diminished capacity contribution in the sloping region (>0.1 V) relative to bare HC. The nearly identical capacity contributions in the plateau region (0.001–0.1 V) for bare HC and PolyHC result in a higher initial discharge capacity for bare HC (402.1 mAh g^–1^) versus PolyHC (360.3 mAh g^–1^), yet the ICE exhibits the converse trend. To evaluate the stability of bare HC and PolyHC, an initial cycling regimen of 100 consecutive cycles was performed at a low current density of 0.05 A g^–1^ (Fig. 3b). The reversible sodium storage capacity of PolyHC stabilized at ∼316 mAh g^–1^, which is slightly higher than that of bare HC (∼303 mAh g^–1^). A slight improvement in capacity and stability indicates that the PESF polymer coating fails to reveal its superiority at low current densities. Furthermore, 600-cycle service trials were conducted on bare HC and PolyHC at a current density of 0.1 A g^–1^, as shown in Fig. 3c, Fig. S13 and Fig. S14a. The bare HC exhibited a progressive decay in sodium storage capacity after 250 cycles, whereas PolyHC sustained its superior cycling stability. After 600 cycles, the PolyHC maintained an exceptional capacity retention of ∼91%, significantly surpassing the ∼76% retention observed for the bare HC counterpart. The GCD profiles of PolyHC and bare HC were scrutinized in detail to elucidate the underlying mechanisms of capacity fading (Fig. 3d and e and Fig. S15). Both the sloping and plateau regions of PolyHC exhibited exceptional cyclic stability, as evidenced by near-overlapping GCD profiles throughout cycling. On the contrary, bare HC presented a progressive decline in slope capacity, which can be attributed to the unstable repeated formation of the SEI, contributing to an increase in irreversible sodium loss.

The rate performance is a key indicator for evaluating the stability and fast-charging capability of the HC anode. The rate performance of bare HC and PolyHC was tested in an ester electrolyte environment (Fig. 3f, Fig. S14b and Fig. S16). Bare HC and PolyHC display similar capacity retention at low current densities, but present a stark contrast at high current densities. When the current density is increased to 0.3 A g^–1^, the capacity of bare HC showed a cliff-like decay from ∼239 to 84 mAh g^–1^, while PolyHC still maintained a high capacity of ∼250 mAh g^–1^. Moreover, bare HC fails to service in fast-charging conditions (1.0 A g^–1^); conversely, PolyHC is capable of maintaining satisfactory cycle stability, which a robust SEI on the surface contributes to. To verify the scalability of the PESF polymer coating technology, we validated the rate performance of the PolyHC anode in an ether electrolyte environment (1 M NaPF_6_ in diglyme), as displayed in Fig. S17. PolyHC exhibits more stable rate performance in ether-based electrolytes than in ester-based electrolytes. As a result, PESF polymer coating technology is adaptable to both ester and ether electrolytes. To further evaluate the superiority of the polymer coating technology, we conducted durability and fast-charging capability tests on PolyHC at a high current density of 0.5 A g^–1^ (Fig. 3g and h and Fig. S14c). PolyHC manifests exceptional cycle stability and durability, which achieves electrochemical longevity exceeding 1500 cycles with 82% retention. As a comparison, bare HC without a PESF polymer coating failed to operate stably under these conditions, as evidenced by a precipitous decline in capacity. The minor capacity decrease in the slope region of the GCD curve confirms the robustness and stability of the PESF polymer-derived SEI. Importantly, the feasibility and universality of the PESF coating strategy were rigorously validated across five distinct industrial-grade HCs, consistently enhancing cycle stability while maintaining ∼90% ICE (Figs S18–S22).

### SEI detection

**Figure 4.**
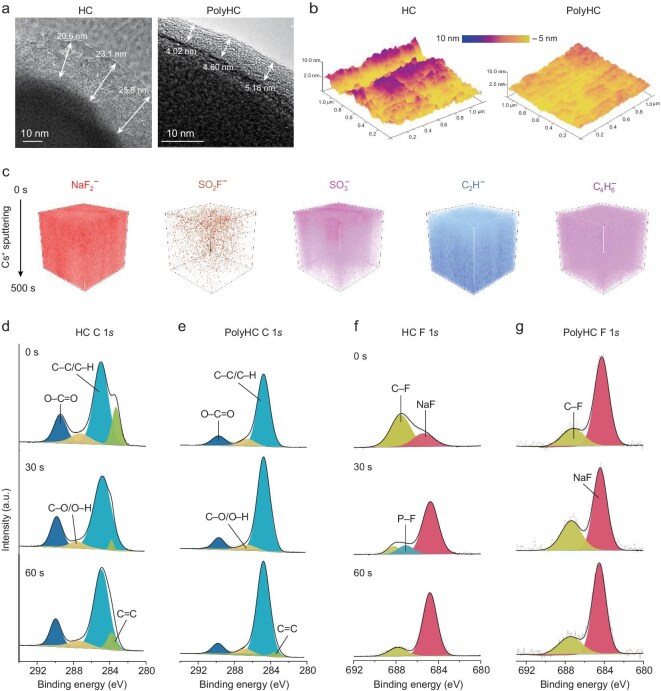
Probing of SEI formed on bare HC and PolyHC surfaces. (a) TEM images of bare HC and PolyHC after SEI formation. (b) AFM images of bare HC and PolyHC surfaces after several cycles. (c) The ToF-SIMS detection results on the PolyHC surface after cycling by etching for 0–500 s in a CS^+^ sputtering environment. (d–g) The XPS C 1*s* and F 1*s* characteristic peaks of (d and f) bare HC and (e and g) PolyHC after 30 cycles at different etching depths.

SEI, a bridge between the electrolyte and the HC electrode, significantly determines the stability and kinetic behavior of SIBs. TEM, atomic force microscopy (AFM), time-of-flight secondary ion mass spectrometry (ToF-SIMS) and X-ray photoelectron spectroscopy (XPS) etching techniques were employed to detect the morphology and composition of the SEI. TEM results showed that a loose and an >25.0 nm thick SEI layer formed on the bare HC surface, while the SEI on the PolyHC surface presented dense, homogeneous and ∼5.0 nm thin features (Fig. 4a). After multiple cycles, the polymer-induced SEI thickness on the PolyHC surface remained consistently at ∼6.0 nm, whereas the SEI thickness on the HC surface progressively accumulated with increasing cycles, reaching a staggering >50.0 nm (Fig. S23). A thin and uniform SEI can simultaneously minimize electrolyte decomposition and lower the sodium ion transport barrier at the interface, thereby substantially reducing irreversible sodium loss (elevated ICE) and boosting interface transport kinetics. The morphology of the SEI on bare HC and PolyHC surfaces is visualized by using AFM technology (Fig. 4b and Fig. S24). The bare HC surface exhibits an uneven, bumpy morphology, which may be attributed to localized irregular decomposition of the electrolyte and dissolution and reconstruction of the SEI. Conversely, the PolyHC surface shows a smooth morphology after cycling due to the PESF polymer layer assisting in the formation of a dense and homogeneous SEI.

To explore the components of SEI, ToF-SIMS is prioritized because it can visualize the fragments within SEI by progressing the etching depth. The SEI formed on the PolyHC surface was detected using a ToF-SIMS sputtering technique with a Cs^+^ target (Fig. 4c and Figs S25–S27). The NaF component maintained a strong signal intensity throughout the entire etching process, contributing to the dissociation of F atoms from the PESF polymer and the reduction products of enriched anions. Moreover, SO_2_F^–^ shows a low relative abundance, whereas its decomposition product, SO_3_^–^, is present at a considerable level, suggesting that the –SO_2_F groups within the PESF polymer may decompose during cycling. Interestingly, the PESF polymer skeleton signal, C_2_H^–^ and C_4_H_6_^–^, keeps a high signal strength, especially in the 100–500 s range (Fig. 4c and Fig. S27). These results indicate that the PESF backbone structure cannot decompose and is involved in the formation of the SEI, hybridizing with NaF. To further verify the detected SEI structure, we performed a comparative analysis of the SEI formed on the bare HC and PolyHC surfaces by using an XPS etching technology (Fig. 4d–g and Figs S28 and S29). Before etching, the C 1 *s* fitting peaks located at 284.8 eV (C–C/C–H), 286.6 eV (C–O/O–H) and 289.8 eV (O–C=O) and O 1 *s* peaking at 533.3 eV (C–O/O–H) and 531.6 eV (C=O/CO_3_) are assigned to ROCO_2_Na/(CH_2_OCO_2_Na)_2_, which are produced by solvent decomposition [18,35]. It should be noted that the PESF skeleton also contains C–C signals. Compared with bare HC (Fig. 4d), the C–C signal intensity on the PolyHC surface cannot change significantly, but the O–C=O and C–O/O–H signals weakened with increasing etching depth, indicating that the polymer coating may reduce solvent decomposition [18,36,37]. Two distinct fitting peaks, positioned at 687.2 and 684.3 eV, are indexed as characteristic peaks of C–F and NaF [18,37–39]. The NaF signal on the PolyHC surface is significantly stronger than that on bare HC, which can be attributed to the dissociation of F atoms from the PESF polymer and the decomposition of anions. To verify the stability of the constructed polymer-induced SEI, we conducted XPS etching experiments on PolyHC after 100 cycles (Fig. S30). Meanwhile, the SEI components after 30 and 100 cycles were compared (Fig. S31). The results indicate that the composition of the polymer-induced SEI exhibits minimal variation, clarifying that a stable SEI has formed on the PolyHC surface. The architecture of the SEI at the PolyHC interface is reconstructed through combined ToF-SIMS depth profiling and XPS etching analysis, with comprehensive compositional evolution detailed in Fig. S32. Owing to the action of PESF, a robust SEI structure hybridized with polymer and NaF was formed on the PolyHC surface, which guarantees its fast-charging capability.

### Interface kinetic probing

**Figure 5.**
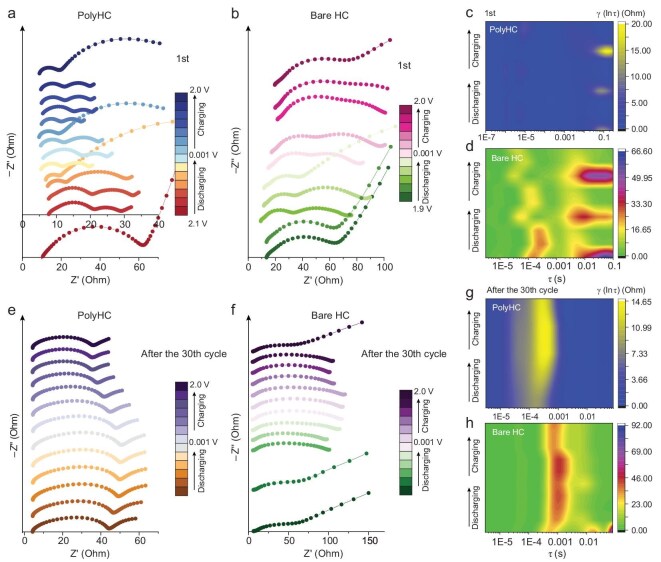
Interface kinetic probing for PolyHC and bare HC. (a and b) The initial *in situ* Nyquist curves of PolyHC and bare HC at the same current density. (c and d) The *in situ* DRT curves of PolyHC and bare HC fitted according to (a) and (b), respectively. (e and f) The *in situ* electrochemical impedance profiles of PolyHC and bare HC after SEI stabilization (cycled for 30 cycles at 0.05 A g^–1^) and (g and h) the corresponding fitted DRT curves.

Electrochemical impedance spectroscopy (EIS) was used to investigate the ion transport kinetic at the interface. *In situ* impedance spectra acquired during the first cycles of the PolyHC and bare HC were employed to reveal the kinetic evolution of interfacial processes during SEI formation (Fig. 5a–d and Figs S33–S35). Initially, PolyHC and bare HC showed similar impedance values as judged by the semi-circular diameter in the high-frequency region. The impedance of both PolyHC and bare HC decreased during the discharge process, which is consistent with previous reports [40–42]. On the contrary, PolyHC exhibited lower impedance than bare HC, which may be attributed to the PESF polymer coating minimizing excessive reduction decomposition of the electrolyte (Fig. S36). During the discharging process, the impedance of bare HC increases progressively, while the resistance of PolyHC cannot continue to rise and remains below 10 Ω. To clarify the impedance of ions passing through the SEI, we performed distribution of relaxation times (DRT) fitting on the *in situ* Nyquist curves (Fig. 5c and d). PolyHC shows stable and low R_SEI_ values, evidenced by highly overlapping DRT curves (Fig. 5c and Fig. S35). Conversely, the R_SEI_ region of bare HC has relatively high impedance, which is caused by uncontrollable electrolyte decomposition on its surface. To investigate the dynamic properties of the stabilized SEI, we conducted *in situ* impedance experiments on bare HC and PolyHC after 30 cycles under the same current conditions (Fig. 5e–h). The impedance of both PolyHC and bare HC remains relatively stable during charging and discharging process (Fig. 5e and f). A stable electrochemical impedance of ∼40 Ω was achieved for PolyHC, in contrast to the ∼100 Ω observed for bare HC. Meanwhile, the DRT fitting results also demonstrate that the SEI formed on the PolyHC surface is more conducive to ion transport than bare HC, thereby promoting battery dynamic behavior.

### Practical fast-charging capability evaluation

**Figure 6.**
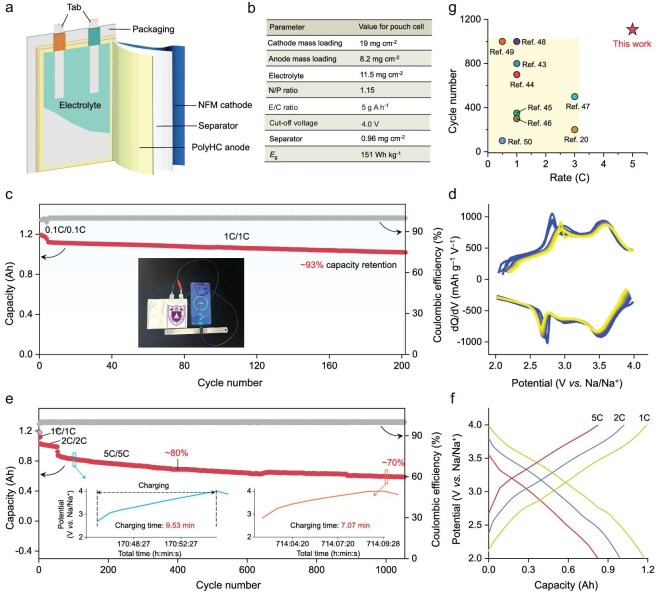
Practical fast-charging capability evaluation. (a) Schematic diagram of the pouch cell, paired with an NFM cathode and a PolyHC anode. (b) The parameter details of this pouch cell component. (c) Cycle behavior of the pouch cell at 1C/1C rate and (d) the corresponding dQ/dV curves. The inset in (c) shows the optical photos of commercially available mobile phones powered by a fully charged pouch cell. (e) Durability and stability testing of pouch cell at 2C/2C and 5C/5C rates, and (f) the corresponding GCD curves. The graphs inset in (e) are the time–voltage curves captured by the pouch cell in 5C/5C fast-charging mode. (g) This work compared with the current state of the art in SIB pouch cells in terms of fast-charging capability.

To demonstrate the practicality of polymer molecular coating technology, a 1.2 Ah pouch cell, matched with a commercially available NFM cathode and PolyHC anode, was assembled (Fig. 6a). This pouch cell shows a competitive energy density of 151 Wh kg^–1^ (Fig. 6b). Interestingly, we utilized a fully charged pouch cell to successfully power an actually available mobile phone and supported its charging to 79%, significantly validating the practicality of polymer molecular coating technology (Fig. 6c, insert). To investigate the stability and fast-charging capability of the pouch cell, we initially pre-cycled it for three cycles and then conducted a cycling test at a 1C charging/1C discharging (1C/1C) rate (Fig. 6c). The pouch cell is capable of operating stably for 200 cycles with a capacity retention ratio of up to 93%, exhibiting superior cycle stability (Fig. 6c). The highly overlapping GCD curves (Fig. S37) and dQ/dV profiles (Fig. 6d) also confirm the stability of the anion redox reaction during the charging and discharging process. Additionally, the fast-charging durability testing of the pouch cell was experimented at a rate of 5C/5C (Fig. 6e). After 50 cycles at 2C/2C, the pouch cell can continuously and stably operate at a high rate of 5C/5C for 1000 cycles, with a capacity retention rate of 80% at 400 cycles and 70% at 1000 cycles, showcasing considerable market competitiveness. The GCD curves reveal that the pouch cell shows almost no capacity degradation at 2C/2C, but undergoes progressive decline in capacity at 5C/5C (Fig. 6f). However, it still maintains continuous cycling, even under the extreme conditions of fast charging and fast discharging. The time–voltage curves (Fig. 6e, inset) further demonstrate that the charging time of the pouch cell can be reduced to <10 min, displaying superior fast-charging capabilities. Our work demonstrates competitive advantages in both cycling longevity and fast-charging capability over state-of-the-art SIBs reported to date (Fig. 6g) [20,43–50].

## CONCLUSIONS

In summary, we have developed a universally polymer-induced SEI strategy that functionally improves the ICE and fast-charging capacity of commercially available HC. The designed PolyHC is capable of minimizing excessive decomposition of the electrolyte at the interface. Benefiting from the strong polarity of the –SO_2_F group, PolyHC successfully induces anion-enriching at the interface and supplies additional F atoms, synergistically contributing to the formation of a ∼5.0 nm thin and robust SEI that combines NaF and polymer. As a result, PolyHC manifests a superlative ICE of 90%, sustains long-term cyclability over 1500 cycles at 0.5 A g^–1^, and delivers exceptional rate kinetics in both ester and ether electrolyte. The assembled 1.2 Ah pouch cell configuration, pairing with NFM cathode and PolyHC anode, achieves fast-charging capability (<10 min) while sustaining 1000 stable cycles, demonstrating compelling practical viability. Importantly, we verified the feasibility of the polymer-induced SEI strategy on another five commercial HC types. This work provides a feasible interface construction strategy for designing SIB fast-charging HC anodes and will inspire more sophisticated interface designs.

## Supplementary Material

nwag025_Supplemental_File
