# Supplementary material for: Polymer-induced solid–electrolyte interphase on hard carbon enabling 5C fast-charging practical sodium-ion pouch cell
Source: Natl Sci Rev. 2026 Jan 19;13(4):nwag025. doi: 10.1093/nsr/nwag025 (PMC12892357; doi:10.1093/nsr/nwag025)
Supplement: nwag025_Supplemental_File [file nwag025_supplemental_file.pdf]

Supplementary Information

Polymer-induced solid electrolyte interphase on hard carbon enabling 5C fast-charging practical sodium-ion pouch cell

Yu Sun,[1,2] Junjie Du,[1] Tianze Shi,[1,2] Daxian Zuo,[1,2] Jiaming Tian,[1,2] Chengrong Xu,[1,2] Bo Peng,[1] Jie Yang,[1,2] Sheng Xu,[1,2] Yiwen Liu,[1] Yu Shi,[1] Haoshen Zhou,[1] and Shaohua Guo[1,2,*]

[1]*College of Engineering and Applied Sciences, Jiangsu Key Laboratory of Artificial Functional Materials, National Laboratory of Solid-State Microstructures, Collaborative Innovation Centre of Advanced Microstructures, Nanjing University, Nanjing 210093, China.*

[2]*Lab of Power and Energy Storage Batteries, Shenzhen Research Institute of Nanjing University, Shenzhen 518000, China.*

*Corresponding authors: E-mails: shguo@nju.edu.cn

Experimental section

Preparation of PolyHC

The ESF monomer molecules (⩾98% analytical purity) were purchased from Nanjing WANQING Chemical Glass ware and Instrument Co., Ltd. Commercially available HC sourced from Bettery BTR New Material Group Co., Ltd. The AIBN inducer is pre-adsorbed on the HC particle surface to drive the interfacial polymerisation reaction. Subsequently, ESF monomers (10 wt%) and Azoisobutyronitrile (AIBN, 99%, 0.3 wt%) initiators were dissolved in a 7:3 volume ratio of N-Methylpyrrolidone (NMP, 99.5%, Sigma-Aldrich) and tetrahydrofuran (THF, 99.9%, Sigma-Aldrich) solvents and stirringly dispersed for 30 min to obtain a homogeneous solution. The HC and modulated solutions were further mixed at a ratio of 1:10 wt% and continuously stirred for 2 h, enabling the ESF monomer molecules to be uniformly distributed around the HC. Subsequently, the above mixture solution undergoes gradient solvent evaporation in a vacuum environment at 313.15K for 30 min, 323.15K for 60 min, 340.15K for 72 h. The product was alternately cleaned with acetonitrile (AN, ⩾99.9%, Sigma-Aldrich) and NMP solvents to remove residual impurities. To avoid unpolymerised or preliminarily polymerised molecules affecting the electrolyte, the cleaned PolyHC was subjected to a filtration process. Finally, the collected material was dried in a vacuum environment at 343.15K, and the PolyHC was prepared. It is noteworthy that the aforementioned process must be carried out under an argon protective atmosphere. Bare HC without any treatment served as the comparison sample.

Characterization

Surface functional groups of bare HC and PolyHC were examined *via* Fourier-transform infrared spectroscopy (FTIR, 450–4000 cm^{-1}) employing a PerkinElmer Spectrum Two-LiTa spectrometer.

Chemical structure and composition were assessed using a Renishaw inVia confocal Raman microscope with a 632.8 nm air-cooled HeNe excitation source. To characterize the crystalline properties of materials, X-ray diffraction (XRD) analysis was conducted utilizing a Bruker D8 Advance instrument equipped with Cu Kα radiation (λ = 1.5418 Å). For compositional depth-profiling within the SEI layer, X-ray photoelectron spectroscopy (XPS) was performed on a Thermo Scientific ESCALAB 250Xi system. Prior to analysis, SEI layers were electrochemically formed on bare HC and PolyHC surfaces through multiple cycles, followed by Dimethoxyethane (DME) rinsing to eliminate residual electrolyte. Surface topography and SEI flatness were investigated through atomic force microscopy (AFM, Bruker Dimension ICON). After cycled, the morphological evolution and elemental distribution of bare HC and PolyHC samples were visualized using a Hitachi SU8000 field-emission scanning electron microscope (SEM). Transmission electron microscopy (TEM, Tecnai F20, Thermo Fisher Scientific) coupled with energy-dispersive X-ray spectroscopy (EDS) elucidated internal carbon-layer structures and SEI thickness. Finally, Time of Flight Secondary Ion Mass Spectrometry (TOF-SIMS, 5 iontof GmbH, Cs^+ source) provided three-dimensional (3D) compositional mapping of the SEI interior.

Electrochemical measurements

The electrode slurry was formulated by homogeneously dissolving optimized PolyHC, Super P conductive additive, and polyvinylidene difluoride (PVDF) binder (mass ratio = 9:0.7:0.3) in an appropriate volume of N-Methyl-2-pyrrolidone (NMP) solvent. This mixture underwent vigorous magnetic stirring at 500 rpm for 5 h to ensure complete dispersion. The resultant slurry was uniformly coated onto pre-cleaned aluminium (Al) foil current collectors using a doctor-blade technique, where the gap height was precisely calibrated to achieve a target active material (PolyHC)

mass loading of approximately 2.0 mg cm^{-2}. Coated foils were subsequently transferred to a vacuum oven and dried at 343.15 K for 48 h to eliminate residual NMP. Circular working electrodes (12 mm diameter) were then punched from the dried foil. Notably, bare HC anode was fabricated following an identical protocol.

SIBs half-cell assembly. In an argon (Ar)-filled glovebox (H_2O/O_2 < 0.1 ppm), CR2032 coin cells were constructed using either bare HC or PolyHC as working electrodes and sodium metal foil as counter/reference electrodes. The electrolyte consisted of 1 M sodium hexafluorophosphate ($NaPF_6$) in ethylene carbonate (EC), methyl ethyl carbonate (EMC), dimethyl carbonate (DMC) (the volume ratio of 1:1:1), with 70 μL aliquots dispensed per cell. Whatman GF/B glass fiber separators were employed to prevent electrical short circuits. The assembled half-cell needs to rest for 12 h for the electrolyte to fully soak the electrodes.

SIBs pouch cell assembly. A commercially available $NaNi_{1/3}Fe_{1/3}Mn_{1/3}O_2$ (NFM) was selected as the cathode material for SIBs pouch cell. The NFM electrode was prepared by dissolving 94% NFM cathode, 3% PVDF, and 3% Super P in NMP, followed by screen coating and drying processes, ensuring an active material loading of 19 mg cm^{-2}. According to the N/P ratio of ~1.15, the active material loading of the PolyHC anode is correspondingly optimized. The separator features a composite structure comprising a polyethylene (PE) substrate coated with a layer of ceramic particles on its surface. The assembly process and environment are similar to those of half-cell.

Characterization. Galvanostatic charge-discharge (GCD) profiles, cycling stability (durability), and rate capability assessments were performed on a NEWARE battery test system. Voltage windows were optimized as 0.001–2.0 V for half-cells (bare HC|Na, PolyHC|Na) and 2.0–4.0 V for pouch cells. Cyclic voltammetry (CV) was conducted using a BioLogic VSP300 potentiostat at scan rates

ranging from 0.1 to 2.0 mV s^{-1}. Electrochemical impedance spectroscopy (EIS) measurements employed a 5 mV AC amplitude across a frequency spectrum of 1.0 MHz to 100 mHz (note: original MHz range corrected to standard mHz for battery EIS). The EIS curves were further fitted by using a distribution of relaxation times (DRT) method.

Molecular dynamics simulation calculations

Molecular dynamics (MD) simulations were conducted in GROMACS 2021.3. Prior to simulation, Gaussian 16 was employed for geometry optimization and frequency analysis of all molecules, with RESP atomic charges and GAFF2 force field parameters derived *via* Multiwfn.[1-4] For the PESF system, CP2K 2025.1 executed structural optimization and periodic charge computations, after which Multiwfn assigned GAFF2 parameters. The simulation protocol involved:[1-4] (1) 10-ns NPT equilibration of the electrolyte at 298.15 K to stabilize volume and molecular distributions; (2) 10-ns NVT pre-equilibration of the full assembly; (3) A 10-ns NVT production phase with trajectory sampling for analysis.

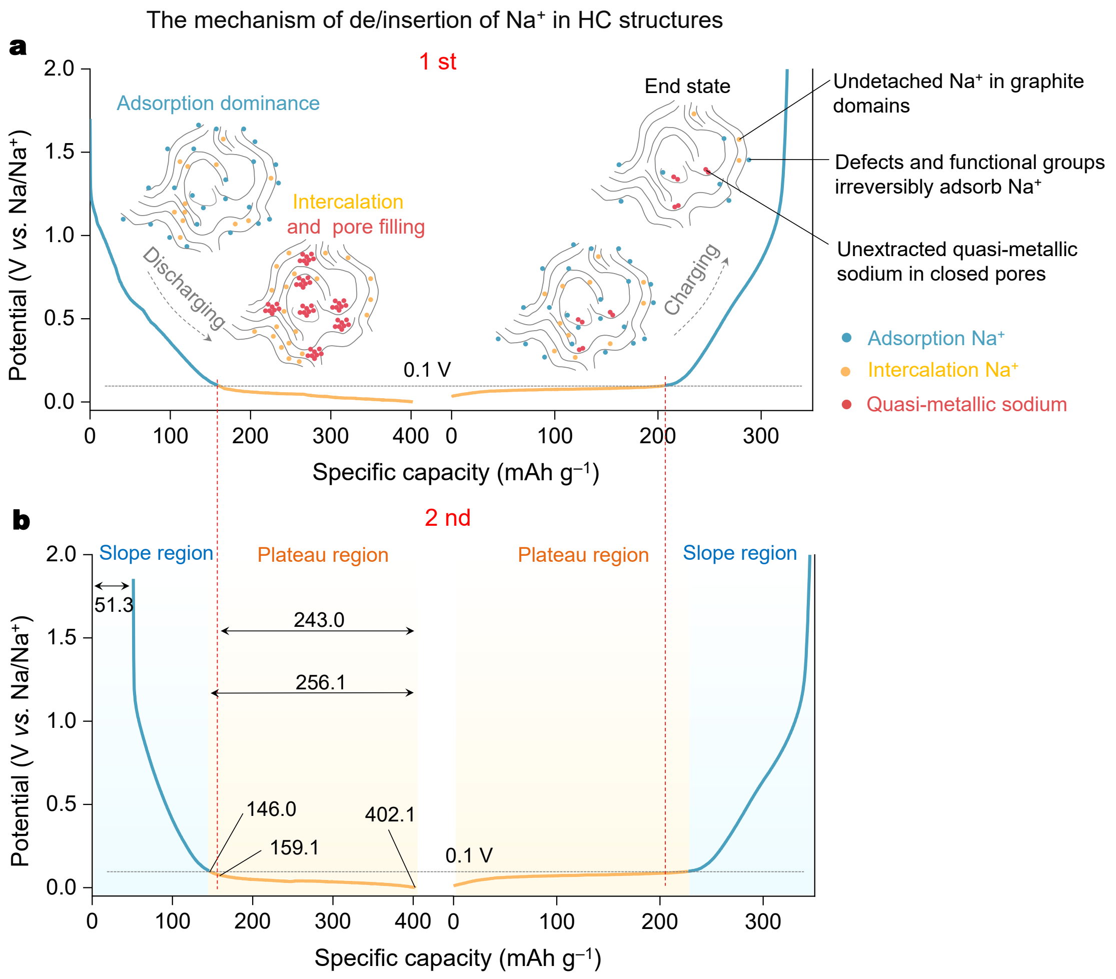

Fig. S1. The mechanism of de/insertion of Na^+ in HC structures. (a) Sodium storage mechanisms of HC in the slope region (> 0.1 V *vs.* Na/Na^+) and platform region (<0.1 V *vs.* Na/Na^+). (b) Comparison of the specific capacity in the slope area and the plateau area for the initial two charge/discharge curves of commercial HC.

Note: We carefully analyzed the initial two GCD curves of commercially available bare HC to identify the primary reasons for its low ICE. The discharge curves of the first and second cycles show similar plateau areas, indicating that the sodium ions inserted and the deposited quasi-metallic sodium are not significantly different, which verifies the stability of the turbine-like structure inside commercial HC. Differently, compared with the first discharge curve, the slope area capacity of the

second discharge curve decreased obviously, indicating that irreversible sodium consumption occurred in this area, which is the primary reason for the low ICE of bare HC. Typically, the slope region is considered to be SEI generated on the bare HC surface and functional groups and defects of HC itself that capture sodium ions. Therefore, how to minimize sodium loss during the SEI formation process or eliminate functional groups and defects is crucial to improving the ICE of commercially available bare HC.

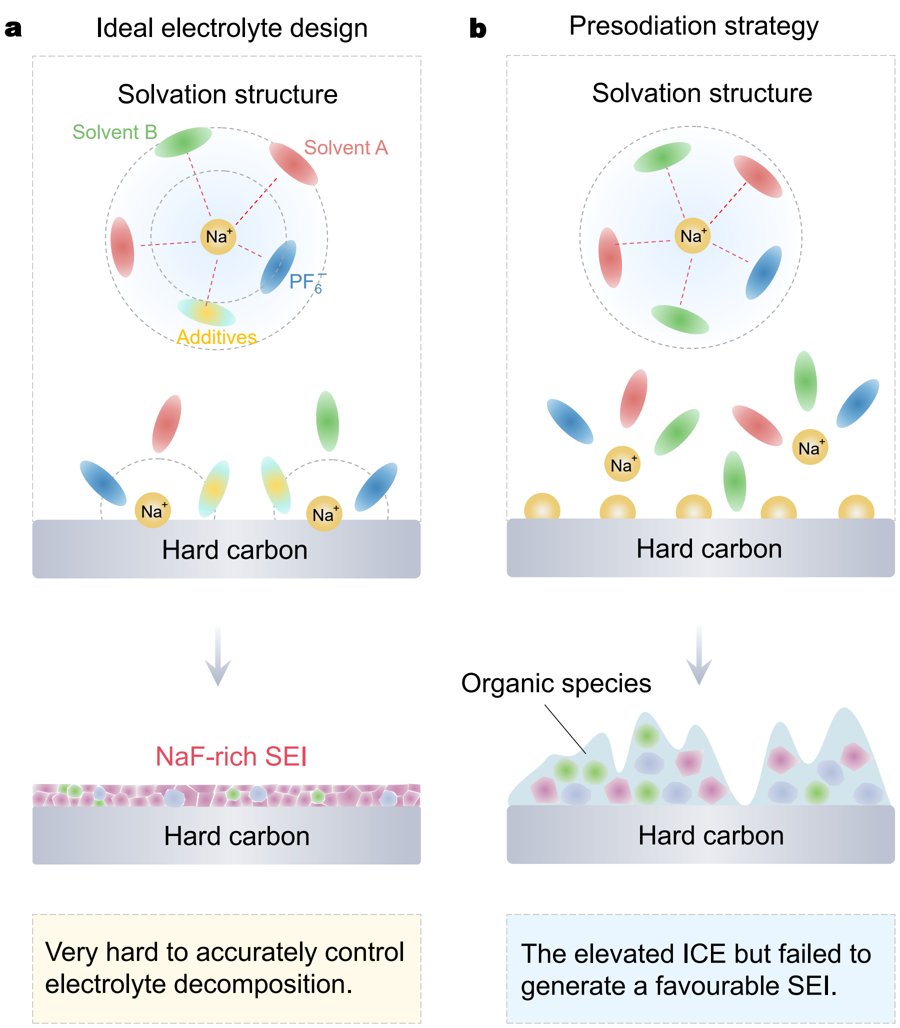

Fig. S2. (a) The ideal electrolyte design for constructing anion-derived SEI. (b) Presodium-treated HC interface improves its ICE.

Note: The ideal electrolyte design relies on forcing anions into the solvated inner shell and preferentially reducing them at the interface to form a NaF-rich SEI, thereby minimizing sodium loss for SEI formation (**Fig. S2a**). Although the electrolyte strategy can reduce sodium loss at the HC interface, it is extremely difficult to simultaneously drive anions into the inner layer of the solvated structure and precisely reduce them at the interface. Another strategy is the presodiation treatment of HC by using organic solutions carrying sodium ions (**Fig. S2b**). The inherent defects and functional groups of HC pre-capture sodium ions by thoroughly soaking it in a sodium-containing organic

solution, enabling that it cannot capture sodium ions in an electrochemical environment, thereby reducing sodium loss at the interface. This method is currently widely used in industrial pre-treatment of HC, but it is limited by its complex operation process and high cost. Moreover, the presodiation technique often fails to induce the formation of a stable and robust SEI at the HC interface, which is unfavourable for subsequent cycle stability, especially under fast charging conditions.

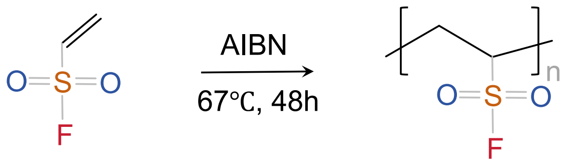

Fig. S3. The polymerization mechanism of ESF monomers. At the action of AIBN initiator, ESF monomers undergo slow *in-situ* polymerization at 340.15K.[5] The above polymerization reaction is employed to design PESF polymer molecular layers coating HC.

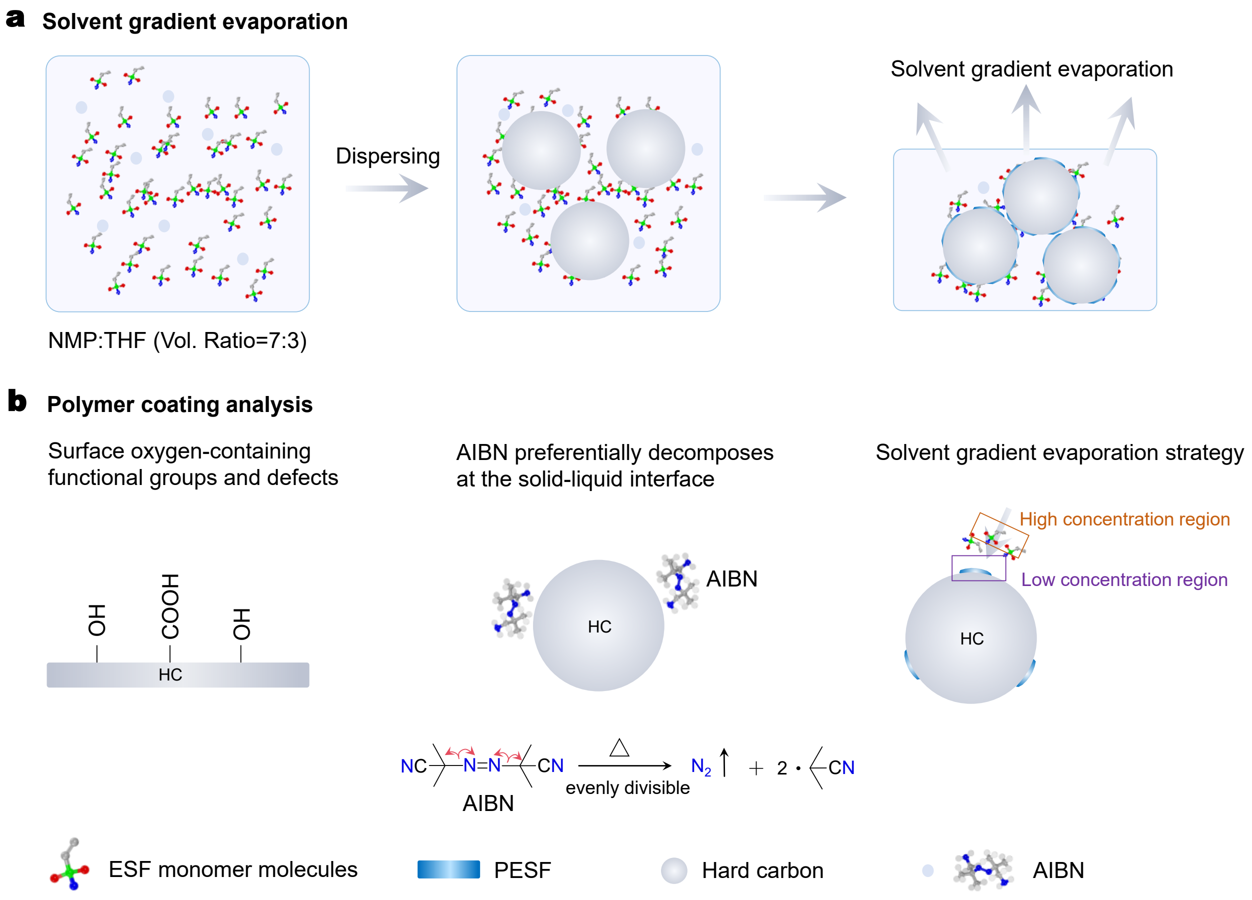

Fig. S4. (a) Solvent gradient evaporation strategy achieves PESF polymer coating HC. (b) Analysis of polymer coating mechanisms. A gradient solvent evaporation process is performed to achieve polymer coating HC.

Note: The AIBN inducer is pre-adsorbed on the hard carbon particle surface to drive the interfacial polymerisation reaction. Subsequently, ESF monomers, minor amounts of AIBN initiator, and the treated HC are uniformly dispersed in an NMP/THF mixed solvent (volume ratio of 7:3) through a continuous stirring dispersion process (**Fig. S4a**). A gradient solvent evaporation process is used to achieve polymer coating HC. The polymer coating mechanism can be summarized as follows (**Fig. S4b**): 1) The functional groups and defects inherent in HC adsorb ESF monomer and AIBN initiator on its surface, enabling *in-situ* polymerization reactions to occur preferentially at the HC interface. 2)

AIBN initiator preferentially decomposes at the solid-liquid interface, which drives the polymerization reaction to occur primarily at the HC interface. 3) The preferential polymerization of ESF molecules at the interface causes the concentration of monomer molecules in this region to be lower than that in the bulk mixed solvent, which favours the movement of more monomer molecules towards the interface. At the same time, as the temperature gradient increases, the THF solvent volatilizes preferentially, causing the concentration of the mixed solution to decrease, which also provides the driving force to ensure that the monomer molecules move towards the interface. To enable that the PESF polymer layer on the HC surface is <4.0 nm, the concentration of ESF monomer molecules is precisely controlled and the temperature gradient is increased gradually.

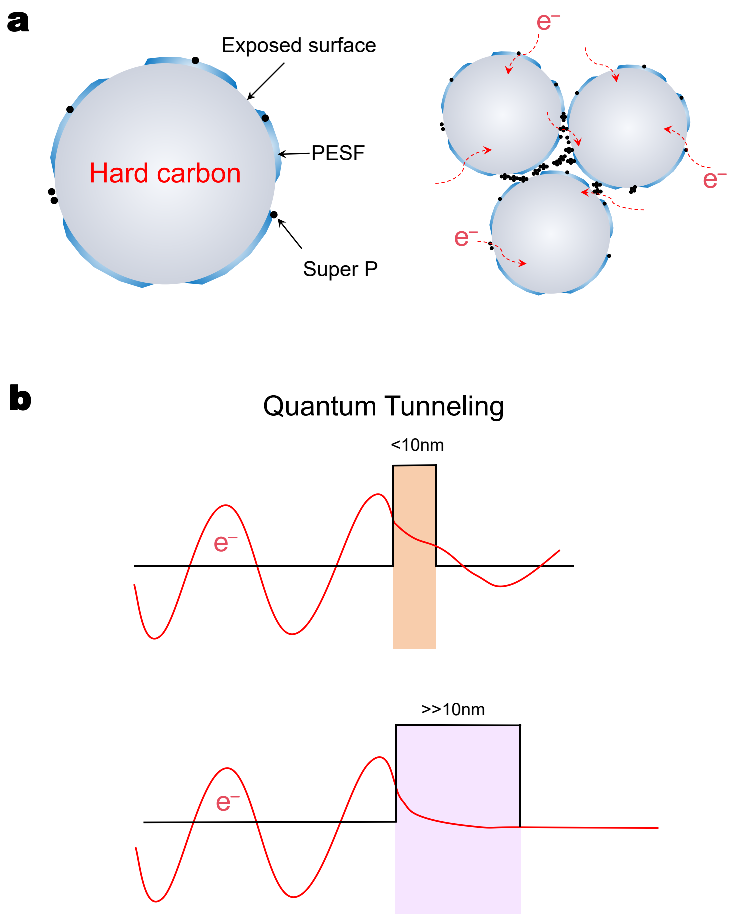

Fig. S5. (a) Electronic transport mechanism of PolyHC integrated electrodes. (b) Schematic diagram of the quantum tunnelling effect.[6]

Note: Considering that the poor conductivity of PESF may affect the overall electron transport capacity of the electrode, we propose the following three factors as explanations: 1) It is extremely hard for PESF polymers to completely coat HC due to the irregular shape and uneven size of bulk commercial HC. The controlled concentration of monomer molecules and the reaction time determine that the polymer chains encapsulate the hard carbon rather than being fully encapsulated. 2) The surrounding activated carbon has good electronic conductivity and can efficiently transmit electrons carried by the current collector. Additionally, the roller pressing electrode process applies a force that causes activated carbon to be locally embedded in PESF, which is conducive to enhancing

the transmission capacity of electrons throughout the PolyHC electrode. 3) When the thickness of the PESF polymer layer is <10 nm, electrons may pass through this barrier into the HC bulk phase (Quantum tunnelling effect).[6] Therefore, the polymer coating may partially hinder the electrons compared to bare HC, but it is not isolated from electron transport inside the particle. Moreover, polymer-induced SEI has thin, homogeneous, and stable characteristics, lowering the interfacial transport barrier for long-term service.

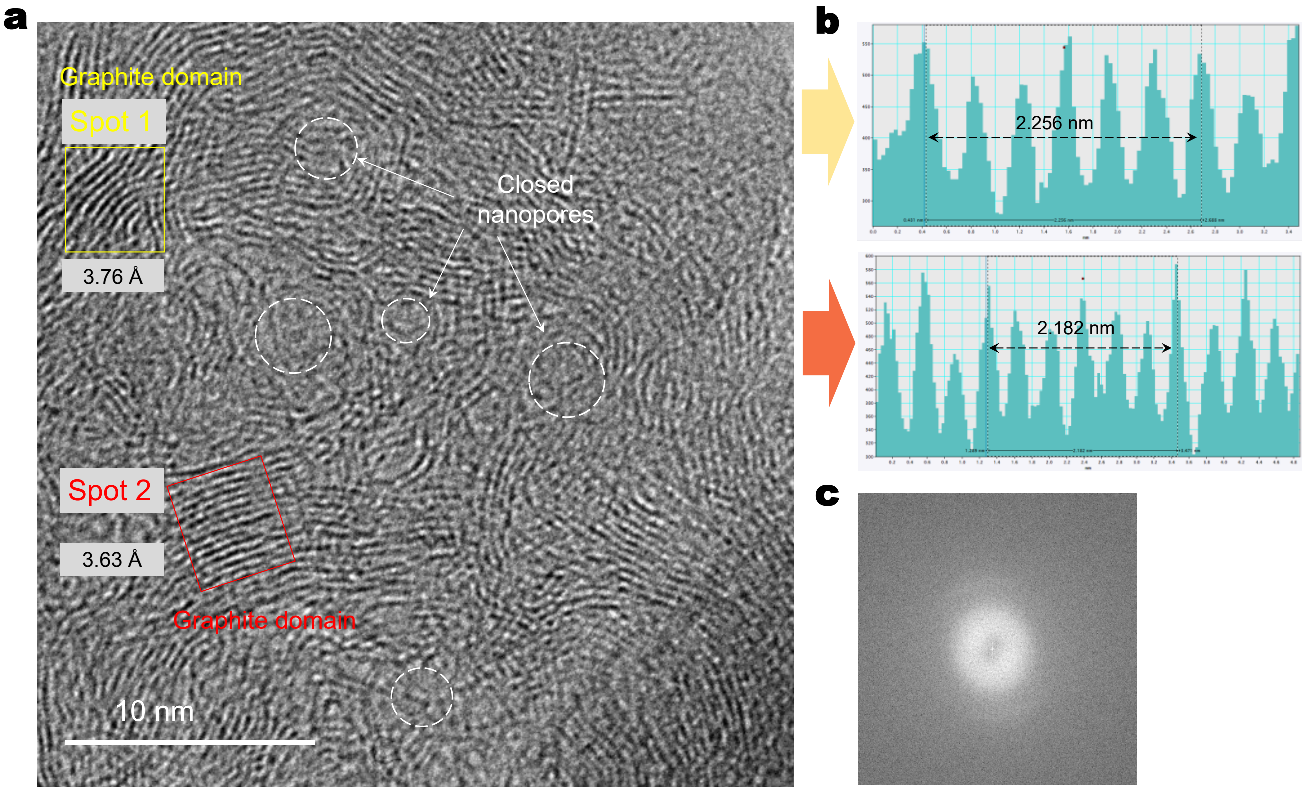

Fig. S6. (a) TEM image of commercial HC bulk phase, (b) its lattice information, and (c) fast fourier transform (FTT) image.

Note: The TEM image of commercial HC presents distinct graphite domain regions and closed nanopore structures (**Fig. S6a**). The graphite domain region corresponds to energy storage *via* sodium ion insertion, while closed nanopores are assigned to the deposition behaviour of quasi-metallic sodium. The interlayer distance of graphite domain was measured to be 3.76 Å at Spot 1 and 3.63 Å at Spot 2 (**Fig. S6b**).

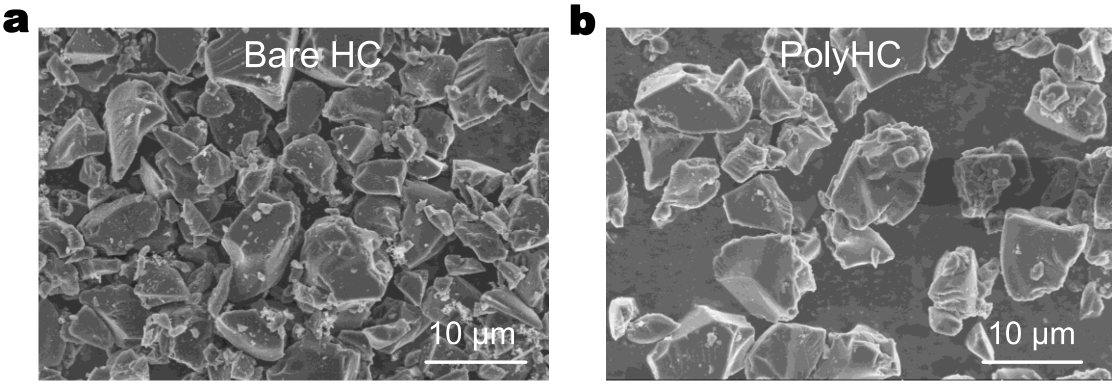

Fig. S7. The SEM images of (a) bare HC and (b) PolyHC.

Note: The selected commercially available HC exhibits irregular shapes with diameters ranging from approximately 2 nm to 10 nm (**Fig. S7a**). After polymer coating, PolyHC shows a similar morphology to HC, indicating that the polymer coating has not altered its inherent morphology (**Fig. S7b**).

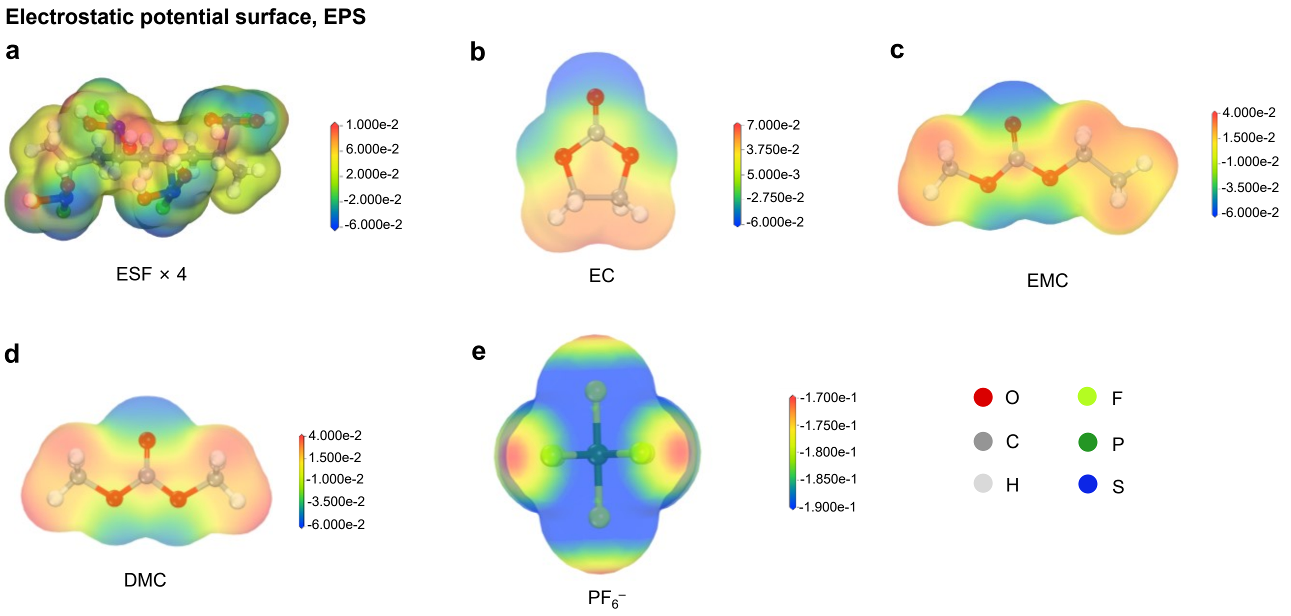

Fig. S8. The electrostatic potential surface (EPS) distribution of (a) ESF (× 4), (b) EC, (c) EMC, (d) DMC, and (e) PF_6^-, respectively.

Note: The EPS distribution of ESF (× 4) presents obvious polarity characteristics, which is attributed to the $–SO_2F$ groups connected to the polymer (**Fig. S8a**). In the sulfonyl group, the sulfur atom forms double bonds with two oxygen atoms (commonly described as having double-bond character). The oxygen atoms possess a very high electronegativity (3.44), whereas the sulfur atom has a lower electronegativity (2.58), resulting in a strongly polar S=O bond. The sulfur atom carries a significant partial positive charge (δ^+), and the oxygen atoms carry significant partial negative charges (δ^-). The $–SO_2F$ group is an extremely strong electron-withdrawing group. This is primarily attributed to: 1) The highly electronegative oxygen and fluorine atoms. These atoms are directly bonded to the sulfur atom *via* covalent bonds and strongly attract electron density. 2) The high oxidation state of sulfur (+6). Sulfur resides in its highest oxidation state, exhibiting a strong inherent tendency to withdraw electrons to stabilize this high oxidation state. This powerful negative inductive effect significantly reduces the electron density of the atom or group directly attached to it (in the case of PESF, the

attached carbon chain), imparting a partial positive charge (δ^+) upon it. Consequently, the PESF polymer layer can effectively induce anion enrichment at the PolyHC interface.

Ethylene carbonate (EC) has a high polarity, the carbonyl oxygen atom region in its ring structure has a negative electrostatic potential, while the methylene region is relatively positive, showing an overall obvious polar character (**Fig. S8b**). Methyl ethyl carbonate (EMC) and dimethyl carbonate (DMC), as chain carbonates, have an electrostatic potential distribution similar to that of EC, with a negatively charged region for the carbonyl oxygen atom and a positively charged region for the alkyl portion, yet with a weaker overall polarity than EC (**Figs. S8c–d**). As an anion, PF_6^- has an overall negative electrostatic potential, and the negative charge is primarily distributed off-domain on the six fluorine atoms, rendering the ion a highly symmetrical surface with negative electrostatic potential that forms strong ion-dipole interactions with solvent molecules. (**Fig. S8e**) Therefore, the polar PESF may interact with the above electrolyte components to affect the solvation structure of the electrolyte at the hard carbon interface. The final results are demonstrated by molecular dynamics simulations.

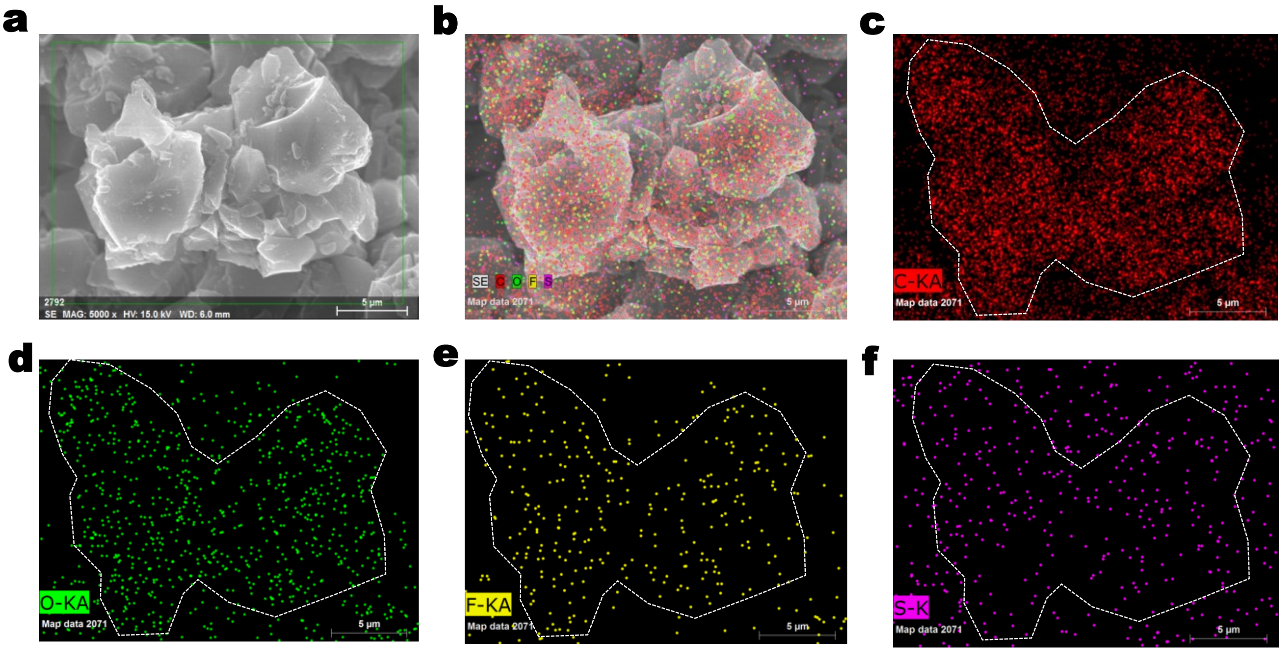

Fig. S9. (a) SEM image of PolyHC and corresponding elemental distributions of (b) overall distribution, (c) C, (d) O, (e) F, and (f) S.

Note: The element mapping results revealed that the characteristic elements of PESF, S, O, and F, are uniformly distributed on the PolyHC surface.

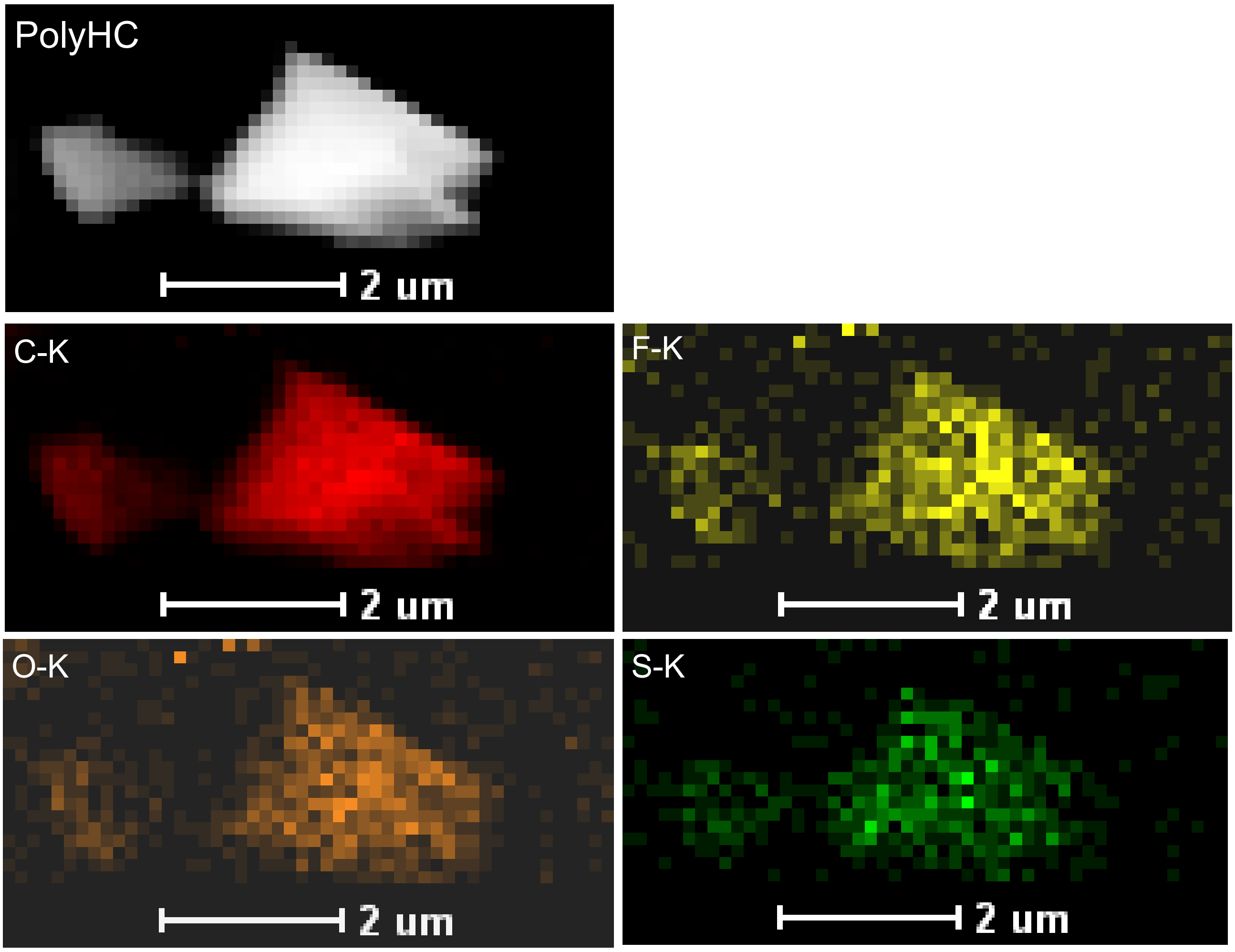

Fig. S10. TEM image of PolyHC and its corresponding elemental distributions.

Note: To further confirm the SEM element mapping results, we conducted TEM element mapping tests on PolyHC. The TEM results obtained showed a clearer elemental distribution than the SEM results, confirming the successful coating of PESF polymer on the PolyHC surface.

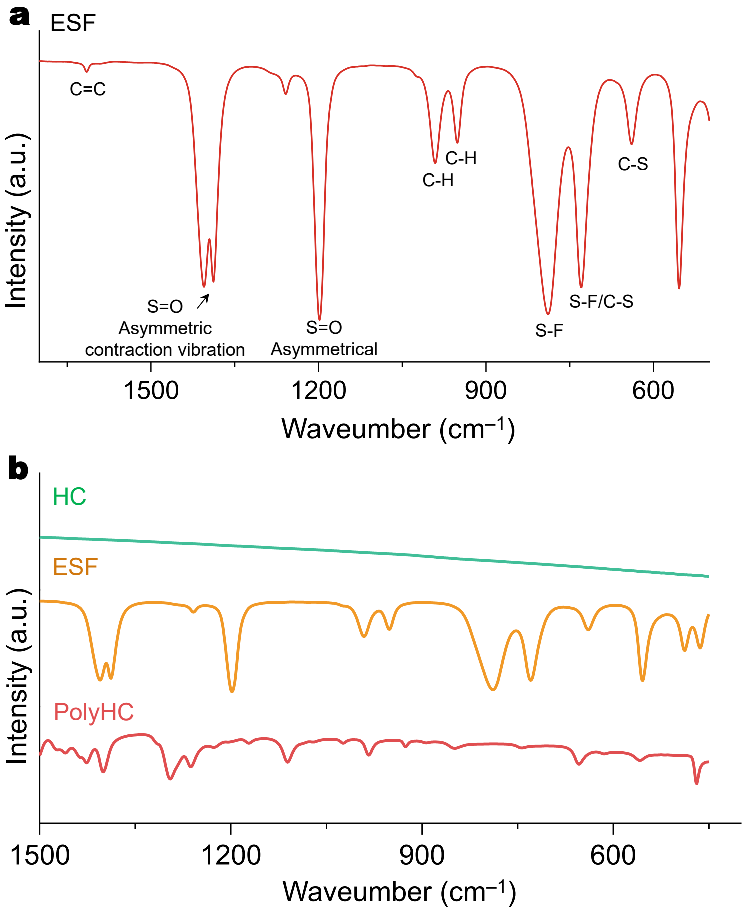

Fig. S11. (a) Fourier transform infrared spectrometer of ESF monomolecules. (b) Infrared spectrum of HC, ESF, and PolyHC, respectively.

Note: The infrared spectrum of ESF monomer molecules is analyzed to identify characteristic functional groups (**Fig. S11a**). To demonstrate the successful coating of PESF polymers, infrared spectra of HC, ESF, and PolyHC are compared (**Fig. S11b**). The commercial HC surface presents without obvious functional group characteristics. Differently, PolyHC exhibits distinct PESF molecular functional group peaks, indicating successful PESF polymer coating, which is consistent with SEM and TEM element distribution.

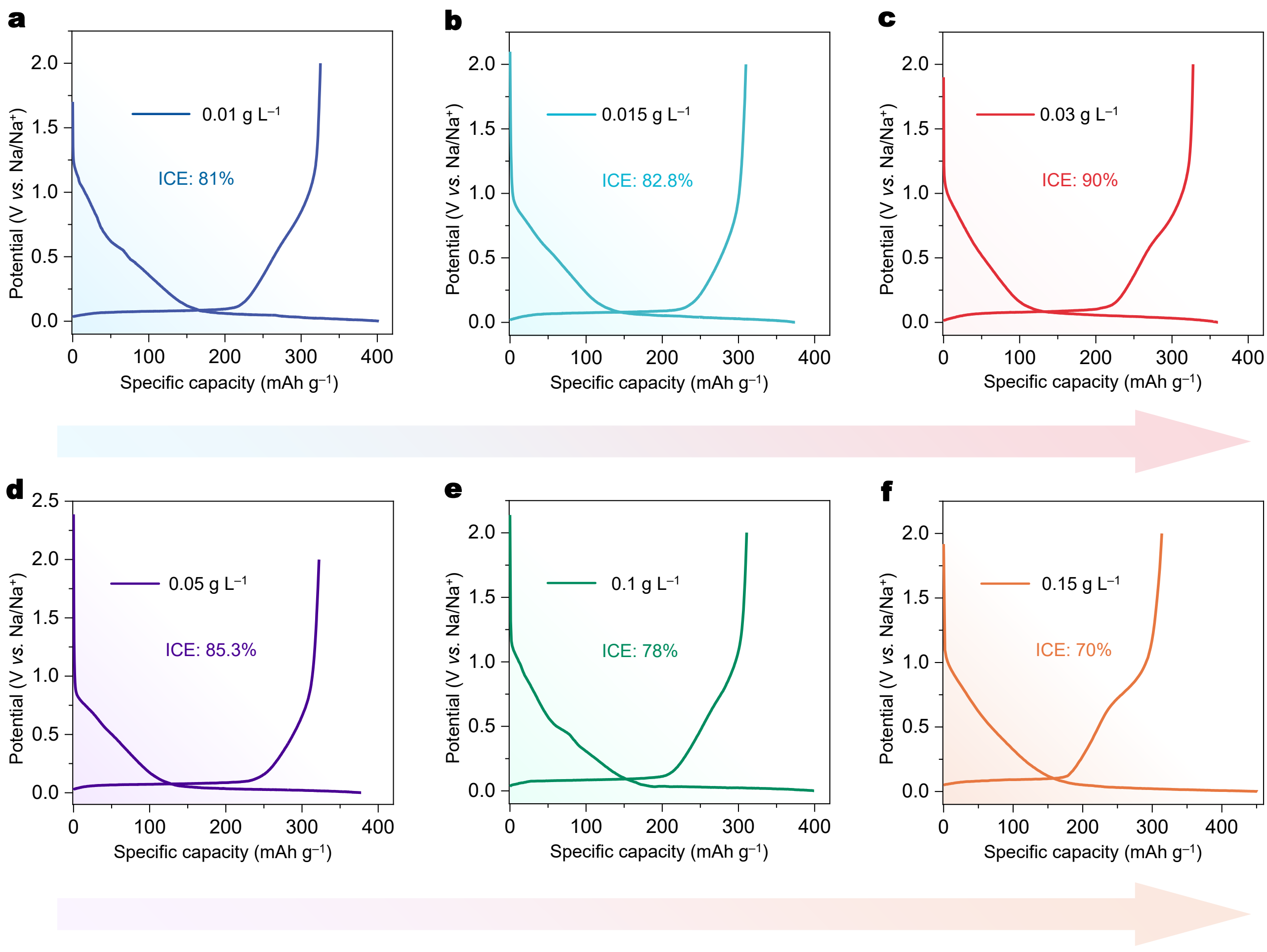

Fig. S12. The concentration of ESF monomers affects the ICE of PolyHC.

Note: To investigate the optimal ESF monomer concentration, we conducted polymerization experiments on commercial HC using various concentrations of ESF monomer solutions (**Fig. S12**). To avoid the influence of particle size, we selected the same commercial HC for the polymer coating experiment. For this HC, an ESF monomer concentration of 0.03 g L^{-1} can achieve a high ICE of 90%. Low concentrations of monomer molecules cannot coat HC, resulting in ineffective suppression of electrolyte decomposition. Conversely, high concentrations of monomer molecules result in increased polymer coating thickness, which is unfavourable for electron transport in the overall electrode and increases interfacial ion transport kinetics. Moreover, PESF polymers itself may consume sodium ions, leading to a lower ICE for PolyHC. Therefore, it is crucial to select

different ESF monomer concentrations for coating different HC, especially for ICE and rate performance.

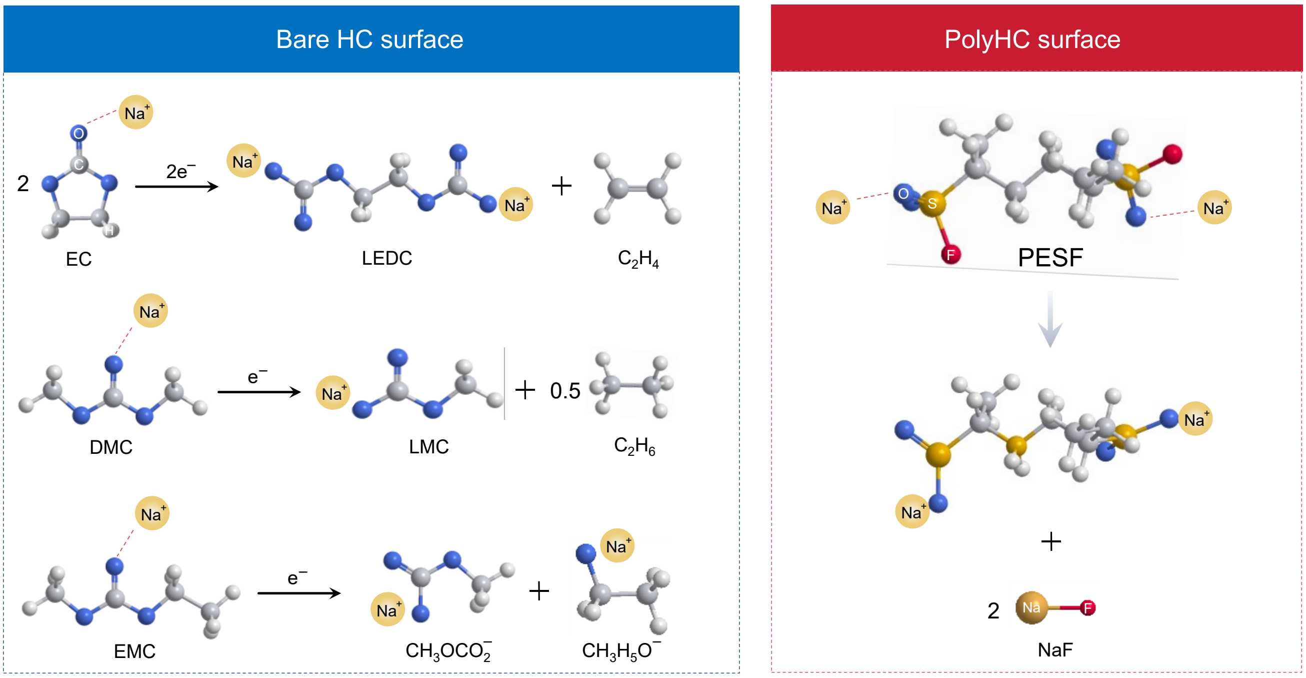

Fig. S13. The probable decomposition mechanisms of bare HC and PolyHC surface in the 1M $NaPF_6$ in EC/EMC/DMC electrolyte.

Note: As the HC surface cannot be treated, the solvent and salt in the electrolyte will decompose on its surface. We have provided the decomposition processes for different solvents, EC, DMC, and EMC.[7] The organic components produced by solvent decomposition exist as part of the SEI, which is not conducive to the formation of a robust SEI and reduces interfacial ion transport kinetics.[7] Owing to the coating of PESF polymer, the electrolyte decomposition behavior on the PolyHC surface differs from that on HC. The $–SO_2F$ group within PESF may be dissociated to release F atoms in a low-potential reduction environment, which is conducive to the formation of SEI enriched with NaF components.

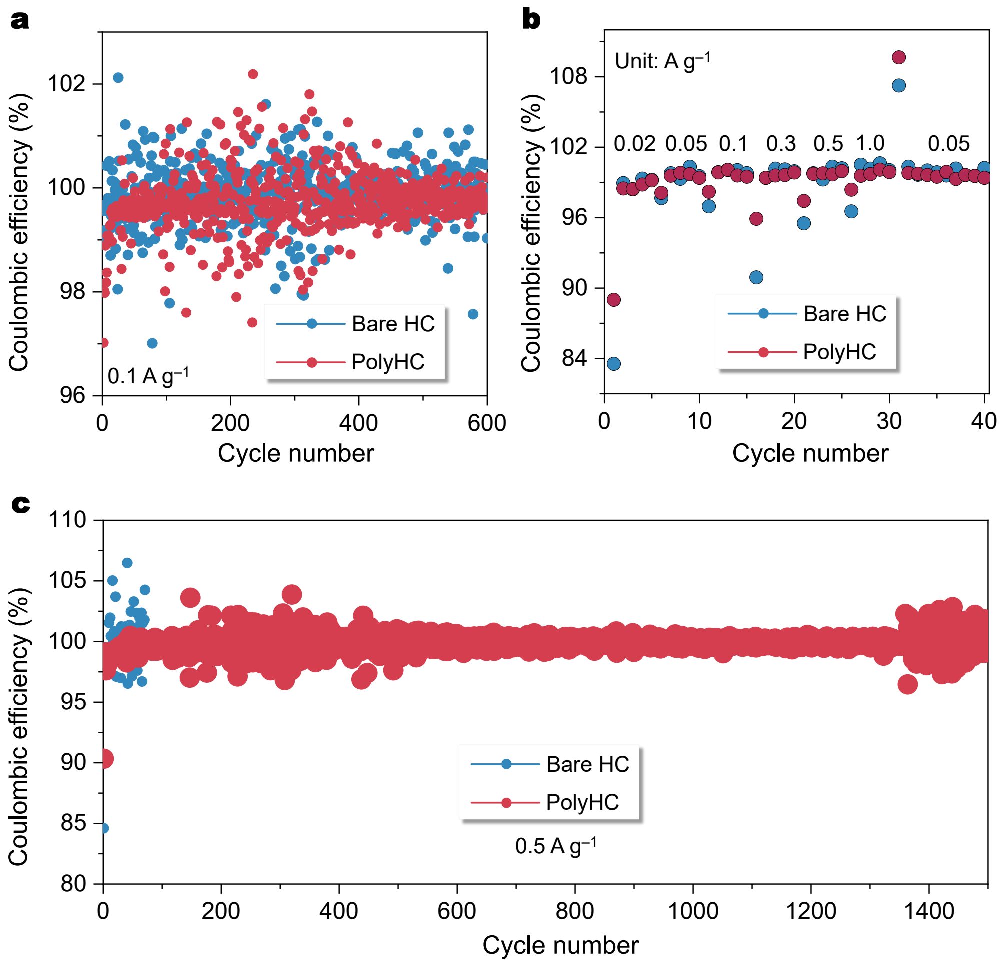

Fig. S14. (a) The ICE of bare HC and PolyHC cycled 600 cycles at 0.1 A g^{-1}. (b) The ICE of bare HC and PolyHC at different rates. (c) The ICE of bare HC and PolyHC in >1500 cycles long-term cycling at 0.5 A g^{-1}.

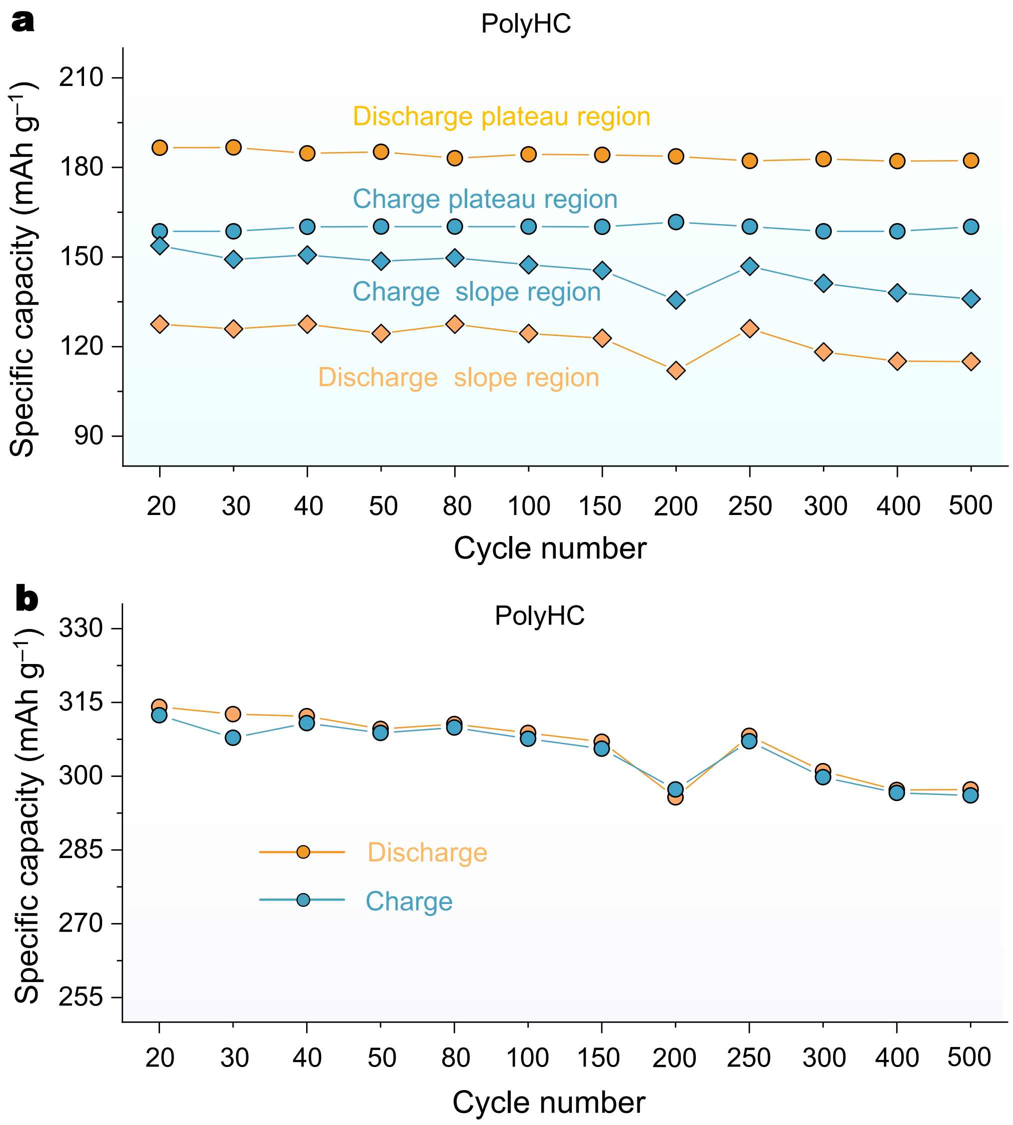

Fig. S15. (a) Analyze the capacity contribution of the slope area and plateau area in the charge and discharge curves. (b) PolyHC capacity variation from 20 to 500 cycles at 0.1 A g^{-1}.

Note: The charging and discharging curves for PolyHC correspond to a plateau area where the capacity remains almost unchanged, which is attributed to the stability of the internal turbine structure of HC (**Fig. S15a**). Meanwhile, the slope area capacity can also remain relatively stable. Charging and discharging capacity contributions are almost identical, indicating that PolyHC remains stable during long-term cycling (**Fig. S15b**).

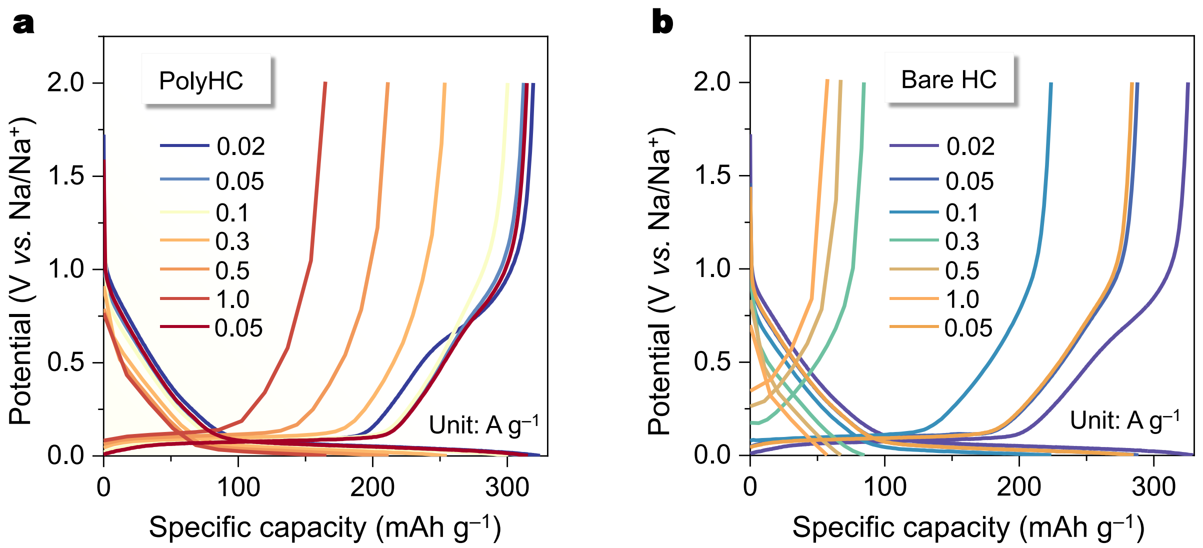

Fig. S16. The GCD curves of (a) PolyHC and (b) bare HC at different rates.

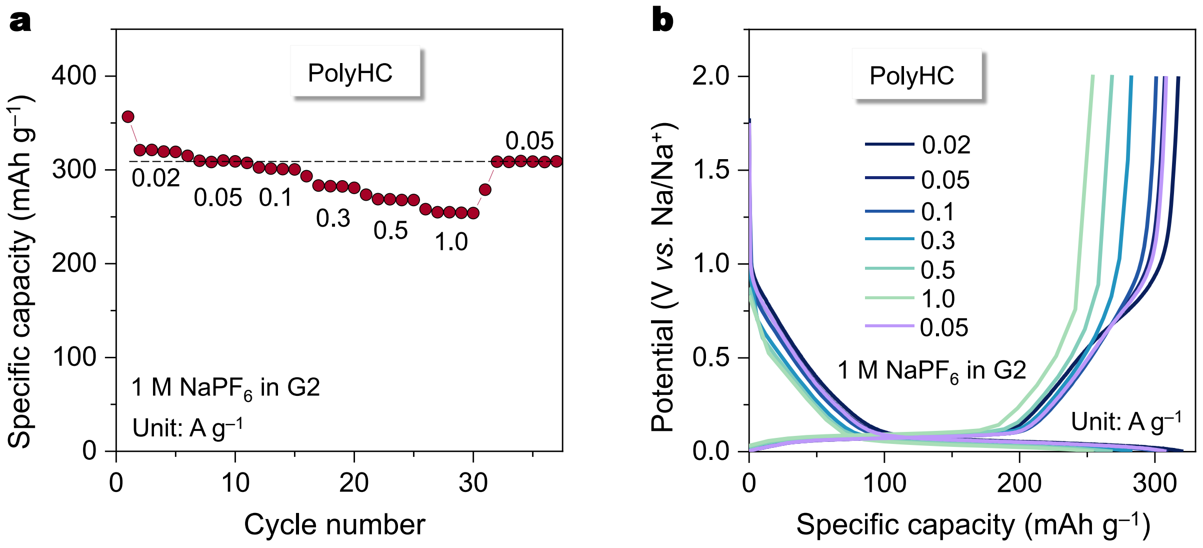

Fig. S17. (a) The rate performance of PolyHC in ether electrolytes, 1M $NaPF_6$ in G2, and (b) its corresponding GCD curves.

Note: To verify the feasibility of polymer-coated PolyHC electrodes in ether electrolytes, we conducted a rate performance testing for PolyHC in 1M $NaPF_6$ in G2 electrolyte (**Fig. S17**). When the current density continues to increase, the capacity of PolyHC progressively decays, but this decreasing trend is better than that in ester electrolytes. Even at a high current density of 1 A g^{-1}, PolyHC still maintains a competitive reversible specific capacity of ~255.3 mAh g^{-1}. When the current density was restored to 0.05 A g^{-1}, the reversible specific capacity of PolyHC recovered to its original value at the identical current density. The aforementioned test results demonstrate that PolyHC maintains excellent rate capability even in ether-based electrolytes, confirming that the polymer coating technology simultaneously exhibits effectiveness in both ester-based and ether-based electrolytes.

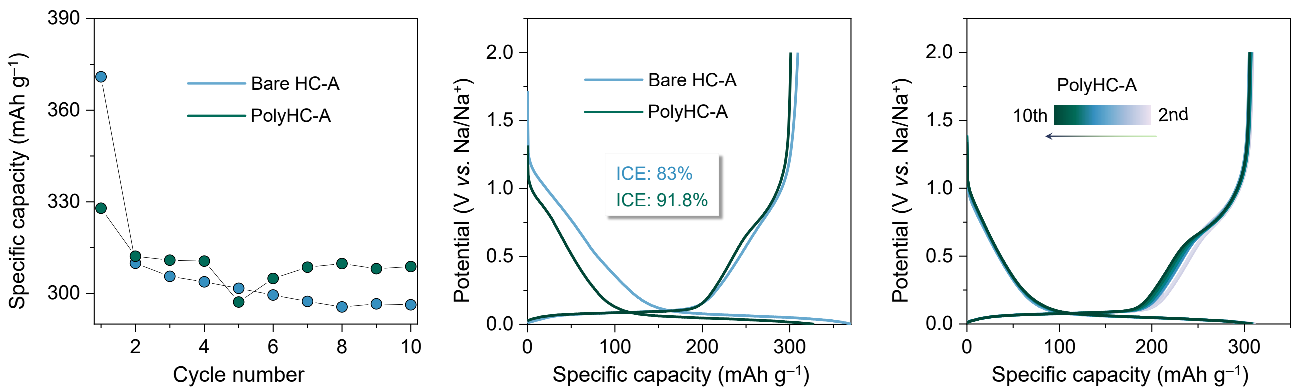

Fig. S18. The cyclic performance of bare HC-A and PolyHC-A, the GCD curve of their initial cycle, and the GCD curves of PolyHC-A. The cycling performed in 1M $NaPF_6$ in EC/EMC/DMC electrolyte at 0.05 A g^{-1}.

Note: To verify the scalability of the polymer coating technology, we conducted polymer coating experiments on another five types, named PolyHC-A, PolyHC-B, PolyHC-C, PolyHC-D, and PolyHC-E, and systematically investigated their electrochemical properties. **Fig. S18** shows the cycling performance of Bare HC-A and PolyHC-A at 0.05 A g^{-1}. The bare HC-A exhibited a high initial discharge capacity of ~371 mAh g^{-1}, but a low ICE of ~83%, which resulted from severe decomposition of the electrolyte on its surface. After polymer coating, PolyHC-A presented a first-cycle reversible sodium storage capacity of ~327.8 mAh g^{-1} and an elevated ICE of ~91.8%, which can be attributed to the polymer coating greatly minimizing the decomposition of the electrolyte at its interface. The nearly overlapping GCD curves demonstrate that a stable SEI was formed on the PolyHC-A surface after polymer coating.

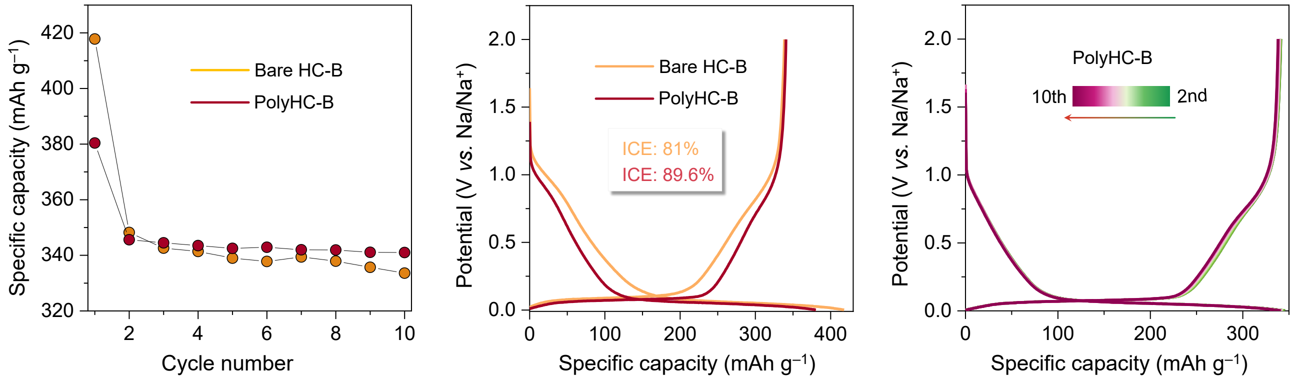

Fig. S19. The cyclic performance of bare HC-B and PolyHC-B, the GCD curve of their initial cycle, and the GCD curves of PolyHC-B. The cycling performed in 1M $NaPF_6$ in EC/EMC/DMC electrolyte at 0.05 A g^{-1}.

Note: PolyHC-B with an ~89.6% enhancement in ICE exhibited a reversible specific capacity of ~380 mAh g^{-1} and enhanced cycle stability. Although bare HC-B has a high initial discharge specific capacity, it is limited by an unsatisfactory ~81% ICE.

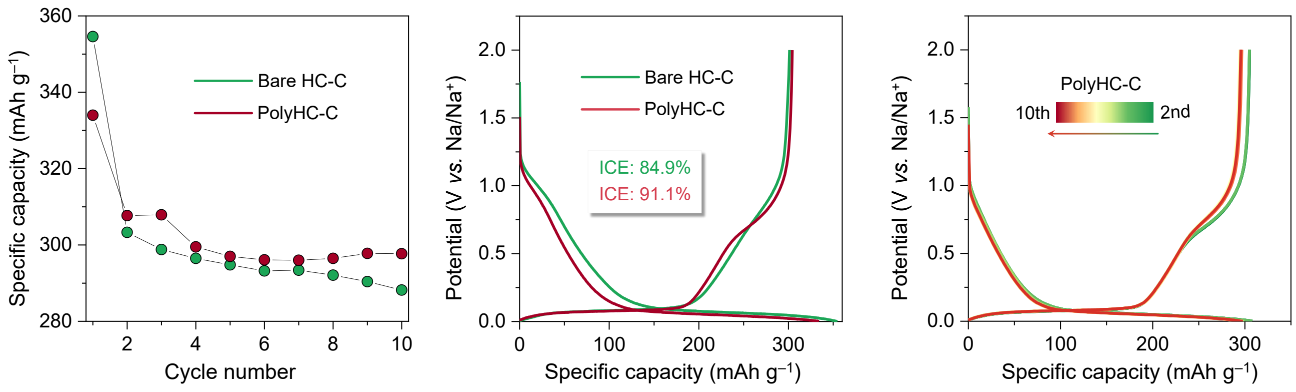

Fig. S20. Cyclic performance of bare HC-C and PolyHC-C, the GCD curve of their initial cycle, and the GCD curves of PolyHC-C. The cycling performed in 1M $NaPF_6$ in EC/EMC/DMC electrolyte at 0.05 A g^{-1}.

Note: After polymer coating, the ICE of bare HC-C improved from 84.9% to 91.1%. At the same time, cyclic stability has also been improved, as evidenced by the highly overlapping GCD curves.

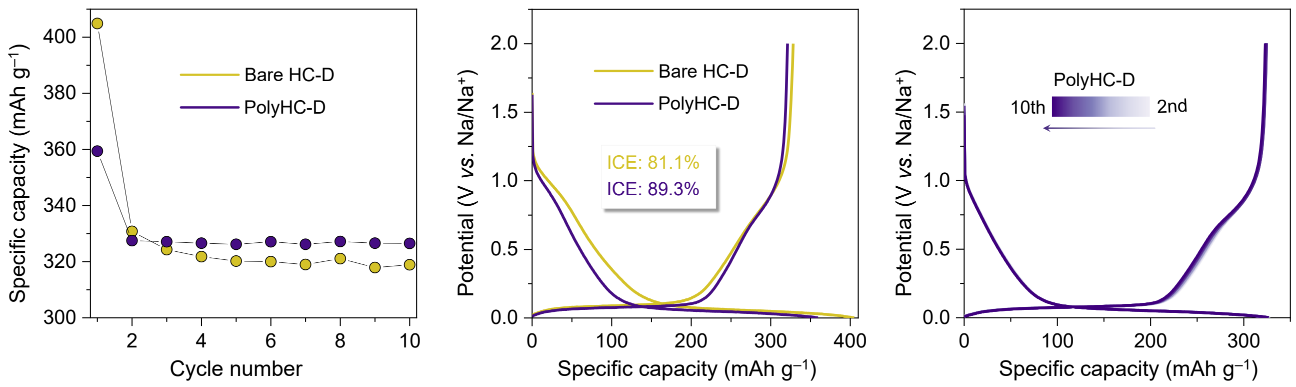

Fig. S21. Cyclic performance of bare HC-D and PolyHC-D, the GCD curve of their initial cycle, and the GCD curves of PolyHC-D. The cycling performed in 1M $NaPF_6$ in EC/EMC/DMC electrolyte at 0.05 A g^{-1}.

Note: Benefiting from the significant suppression of electrolyte decomposition by polymer coating, PolyHC-D displayed a stable reversible specific capacity of ~327 mAh g^{-1} and an improved ICE (from 81.1% to 89.3%), validating the superiority and scalability of polymer coating technology.

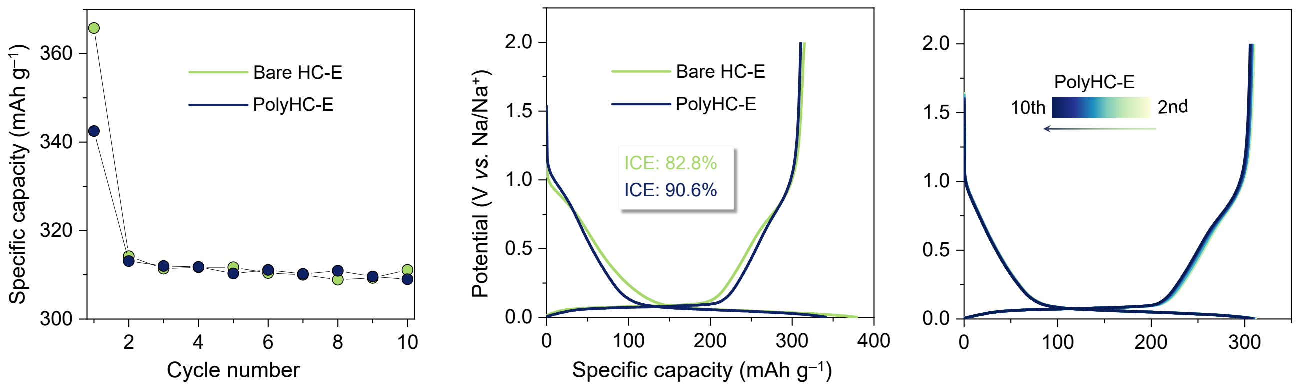

Fig. S22. Cyclic performance of bare HC-E and PolyHC-E, the GCD curve of their initial cycle, and the GCD curves of PolyHC-E. The cycling performed in 1M $NaPF_6$ in EC/EMC/DMC electrolyte at 0.05 A g^{-1}.

Note: Owing to the high stability of the selected bare HC-E, the polymer-coated PolyHC-E and HC exhibited similar stability. However, the surface of bare HC-E cannot control the decomposition of the electrolyte to form a stable SEI, resulting in a low ICE of ~82.8%. Conversely, PolyHC-E presents an improved ICE (90.6%) and superior cycle stability.

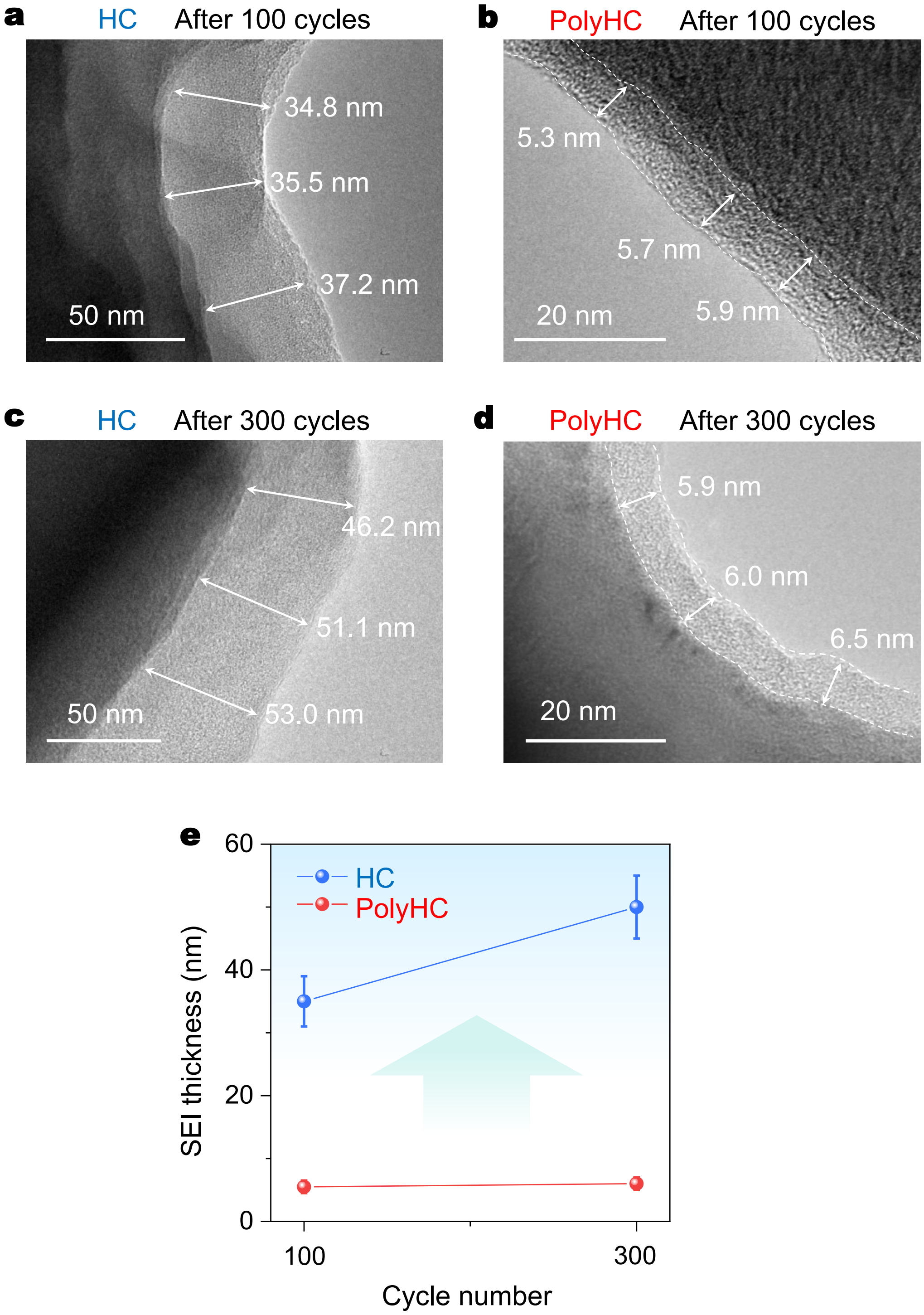

Fig. S23. TEM image of SEI formation on the (a, c) HC and (b, d) PolyHC surfaces after 100 cycles and 300 cycles, respectively. (e) SEI thickness varies with cycle progression.

Note: **Figs. S23a and c** present the TEM images of the HC after 100 and 300 cycles, respectively. The SEI thickness on the HC surface reached ~35.0 nm after 100 cycles (**Fig. 23a**). This accumulation continued to increase, even exceeding 50.0 nm after 300 cycles (**Fig. 23c**), indicating continuous depletion of the electrolyte, which can be attributed to the fact that the unstable interfacial structure exposes fresh hard carbon surfaces exacerbating electrolyte decomposition. Moreover, a thick SEI structure simultaneously reduced ion transport kinetics at the interface and shortened its service life. Differently, the thickness of the polymer-based SEI formed on the PolyHC surface can be stably maintained at ~6.0 nm, even after multiple cycles (**Figs. 23b, d, and e**), demonstrating superior interfacial structural stability.

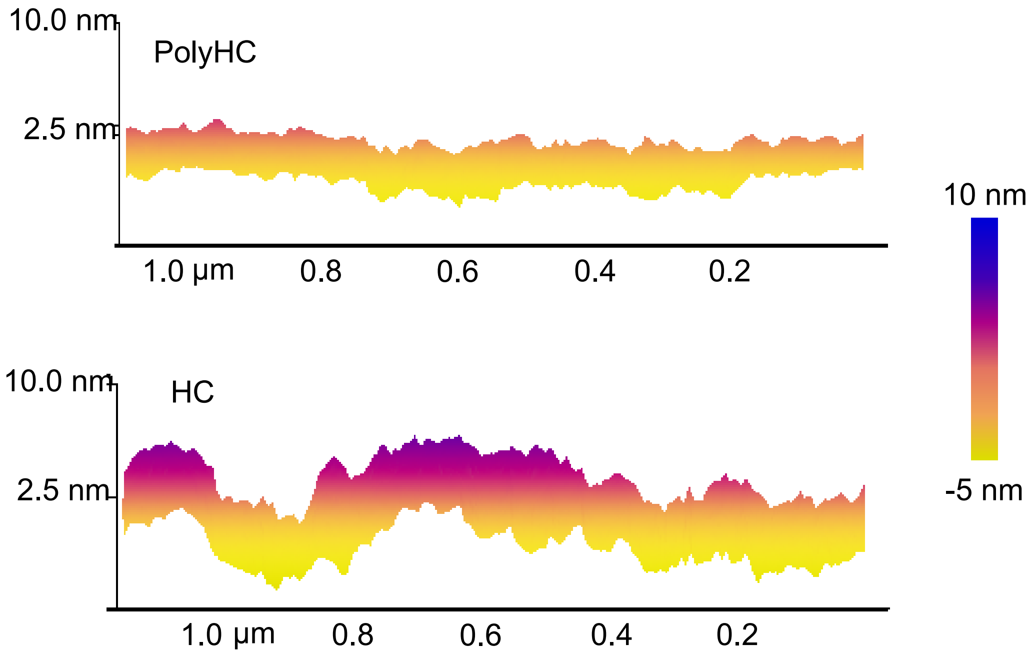

Fig. S24. AFM side images of bare HC and PolyHC surfaces after several cycles.

Note: AFM side images of bare HC and PolyHC reveal that a relatively flat SEI has formed on the surface of PolyHC, while the surface of bare HC exhibits an undulating SEI morphology.

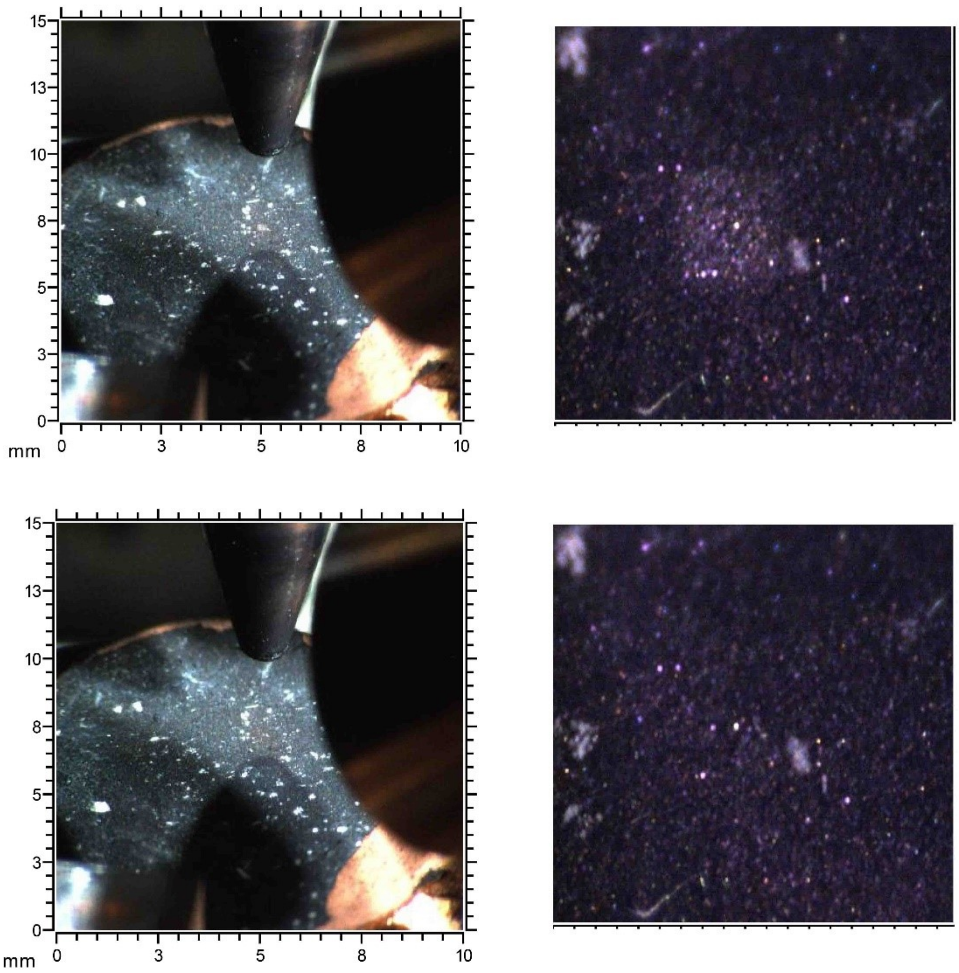

Fig. S25. Visualisation of the ToF-SIMS test environment diagram.

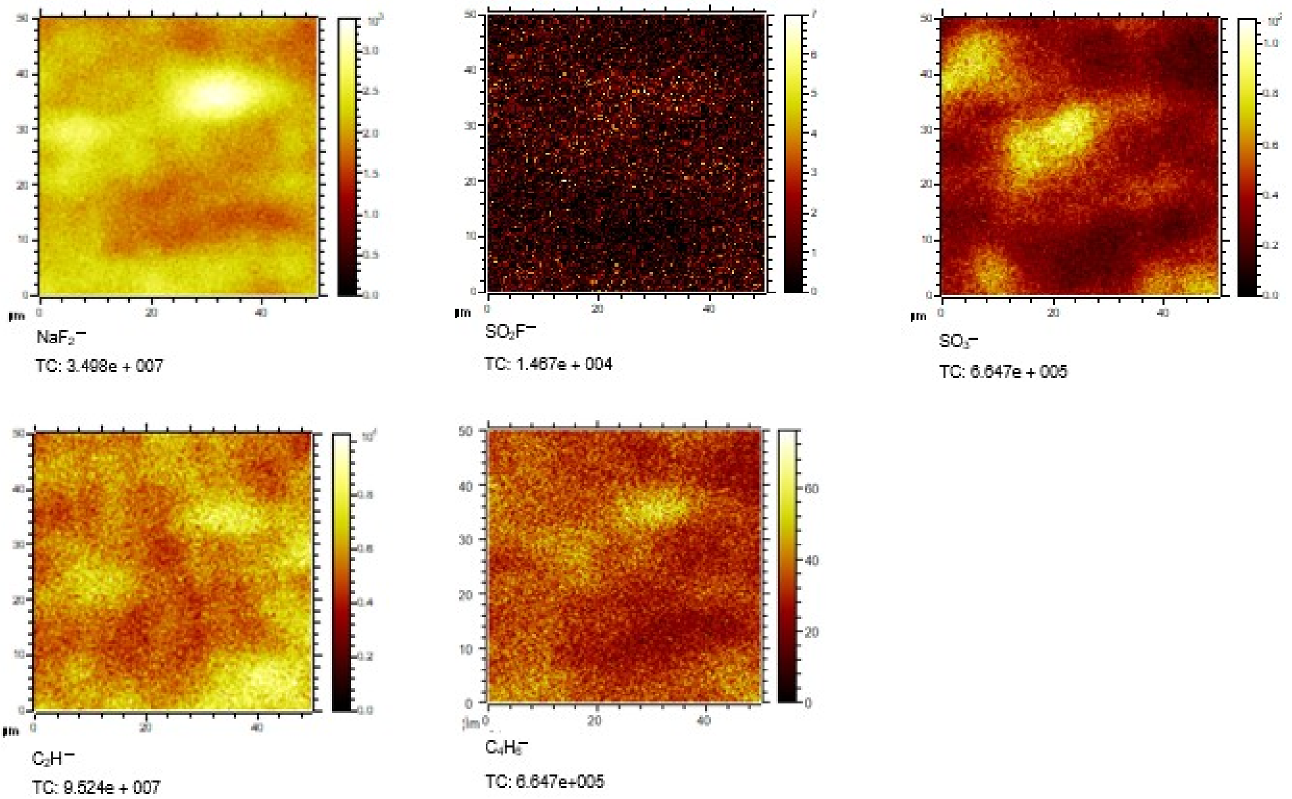

Fig. S26. ToF-SIMS chemical maps of second ion fragments with 500s of sputtering in the cycled PolyHC electrodes.

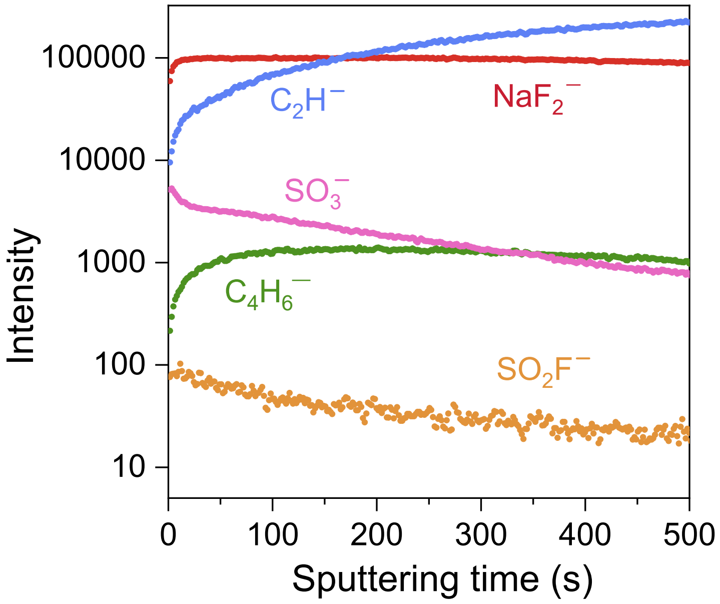

Fig. S27. ToF-SIMS normalized (to maximum) depth profiling of several typical second ion fragments on the cycled PolyHC electrode surface.

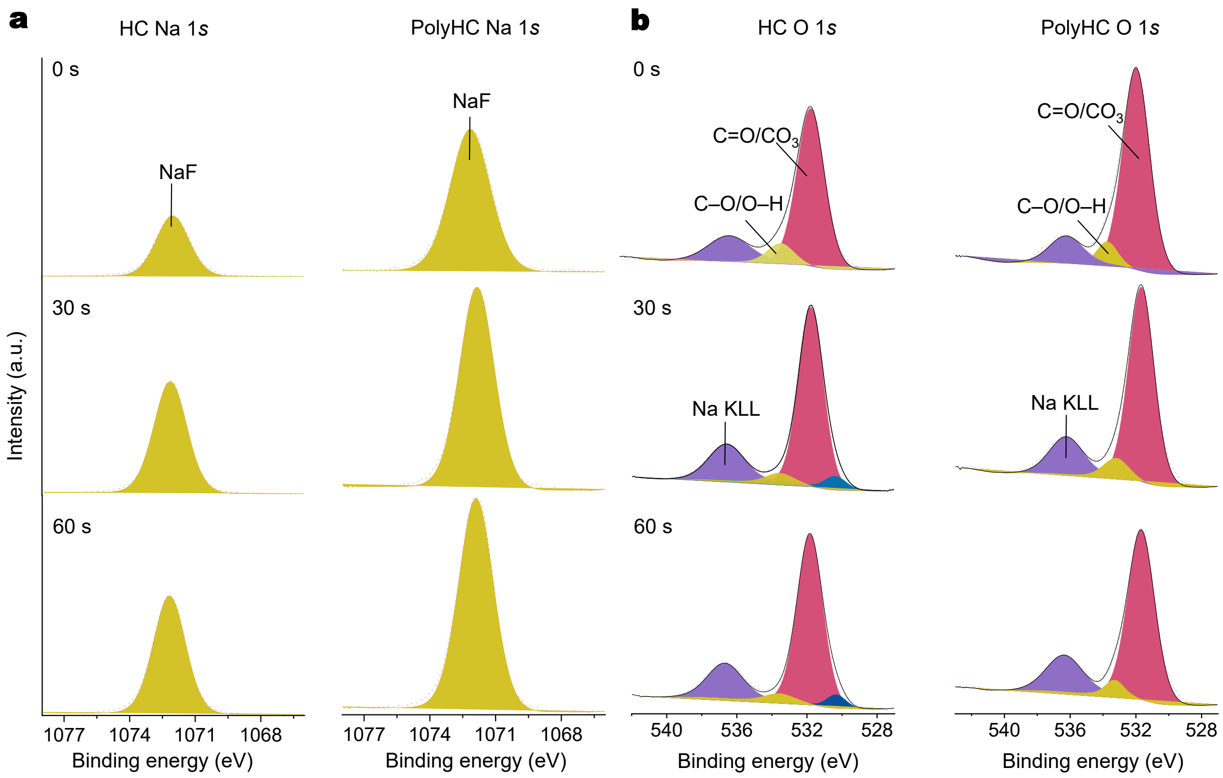

Fig. S28. The XPS Na 1*s* and O 1*s* characteristic peaks of bare HC and PolyHC after 30 cycles at different etching depths.

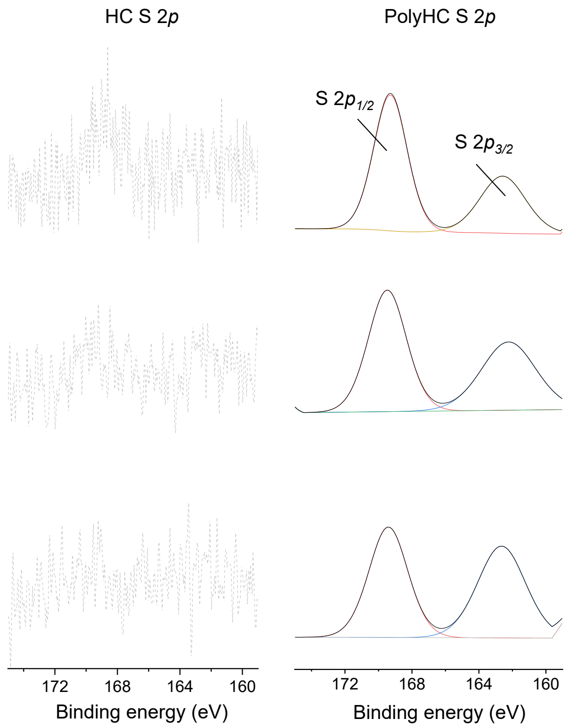

Fig. S29. The XPS S 2*p* characteristic peaks of bare HC and PolyHC after 30 cycles at different etching depths.

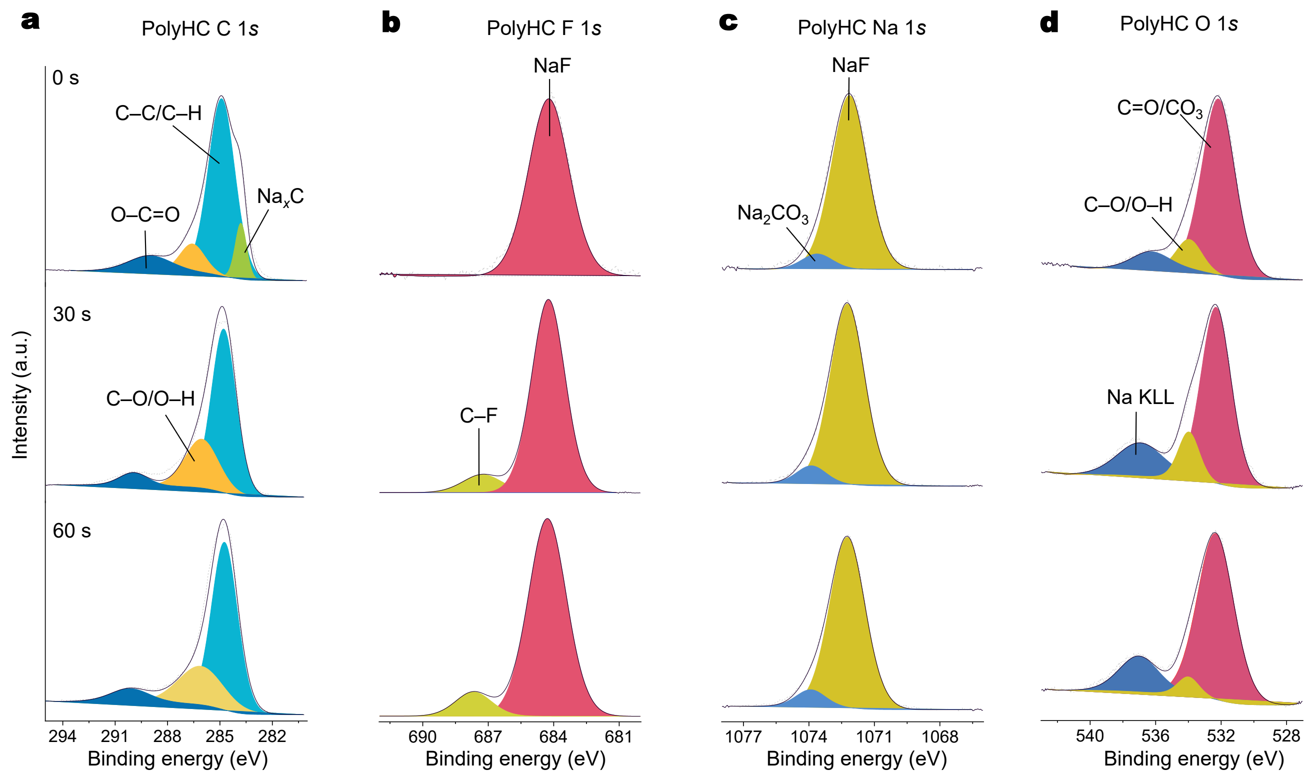

Fig. S30. The XPS (a) C 1*s* , (b) F 1*s* , (c) Na 1*s* and (d) O 1*s* characteristic peaks of PolyHC after 100 cycles at different etching depths.

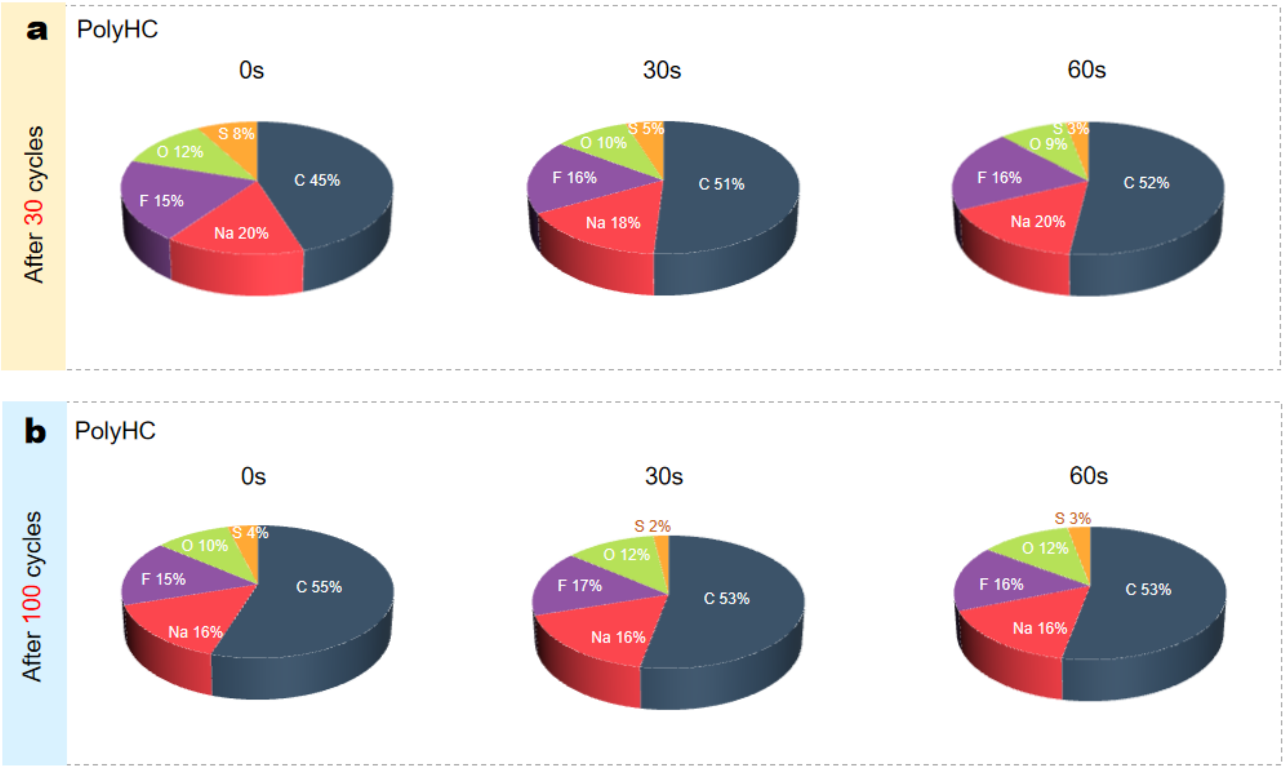

Fig. S31. Percentage ratio of SEI in PolyHC after 30 and 100 cycles, with data derived from XPS fitted at different depths. The data error is controlled within ~5%.

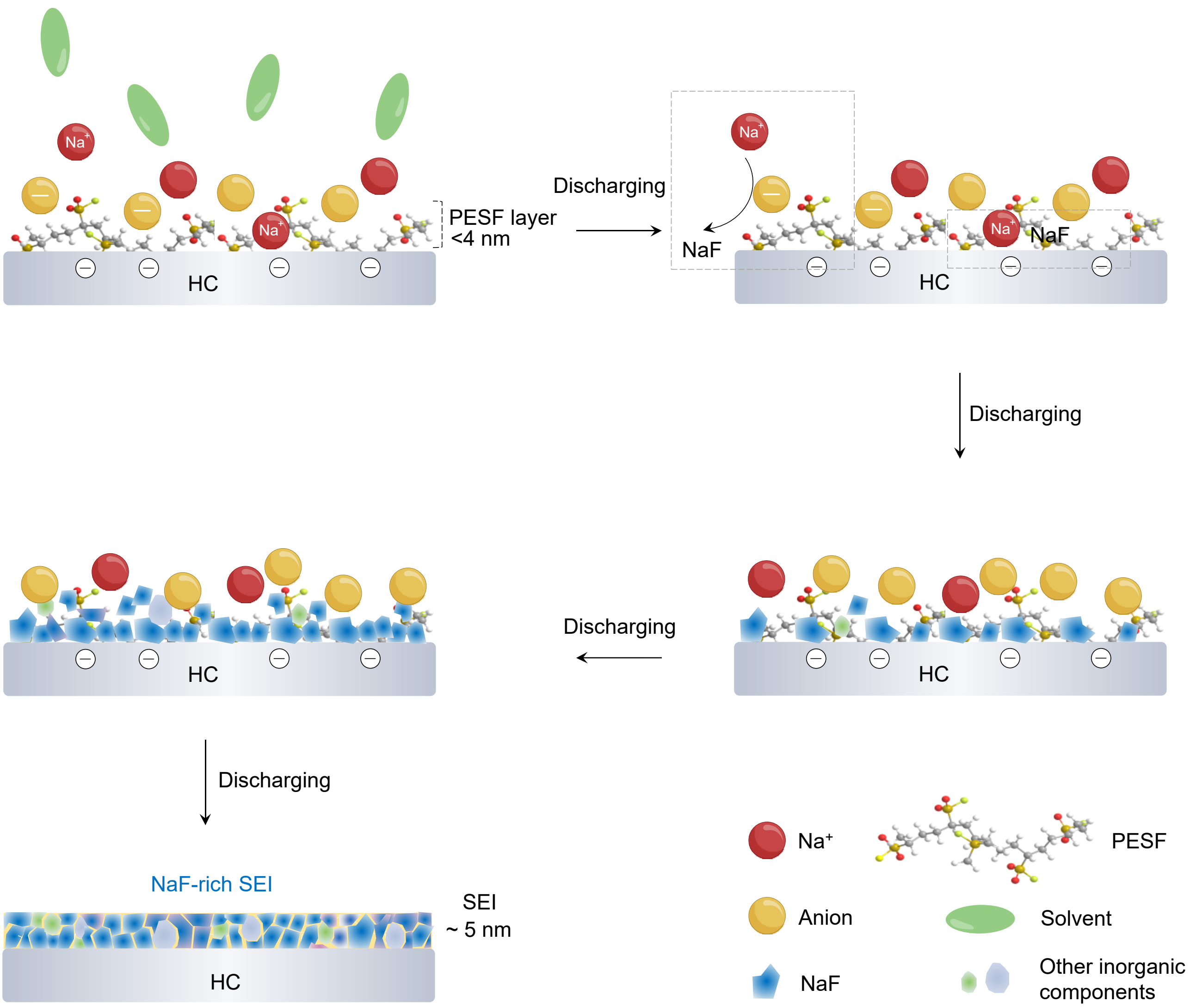

Fig. S32. Schematic diagram of SEI formation on the surface of PolyHC during the discharge process.

Note: According to ToF-SIMS and XPS etching results, we proposed the formation process of SEI on the PolyHC surface (**Fig. S32**). Owing to the –SO_2F groups connected to the PESF polymer, the anions in the electrolyte are absorbed at the PolyHC interface. Anions are reduced under low-potential reduction conditions to form a NaF-rich SEI. Moreover, the –SO_2F groups can also dissociate F atoms to assist in the formation of SEI rich in NaF. It is worth noting that the backbone of the polymer layer remained intact, as evidenced by the ToF-SIMS detection results. Therefore,

NaF is incorporated into the polymer layer and acts synergistically as a component of the SEI. The inherent toughness characteristics of the polymer layer are beneficial for enhancing the stability of the SEI structure, especially at high rates (fast charging conditions). Additionally, owing to the inhibited decomposition of the electrolyte, the thickness of the formed SEI is controlled to ~5.0 nm, which is beneficial for shortening the interfacial ion transport path and minimizing interfacial impedance.

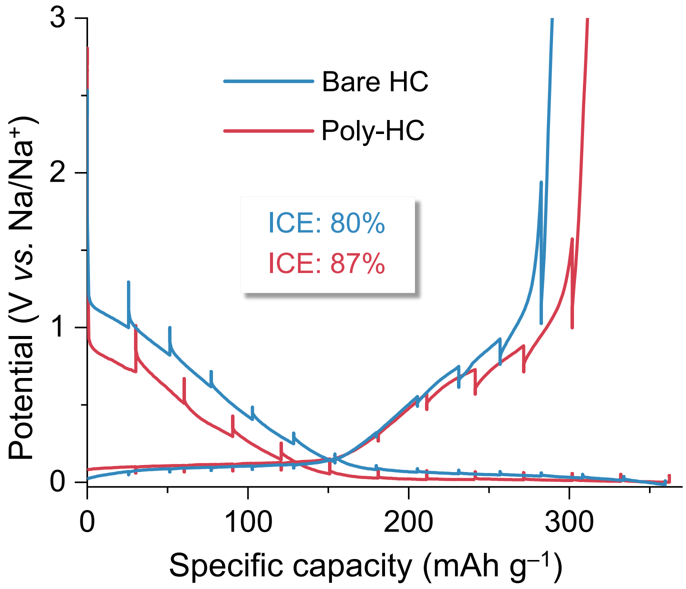

Fig. S33. The initial Galvanostatic Intermittent Titration Technique (GITT) curves of bare HC and PolyHC.

Note: The GITT results also show that, compared with bare HC, PolyHC with polymer coating can effectively reduce the capacity loss in the slope region and improve its ICE (from 80% to 87%).

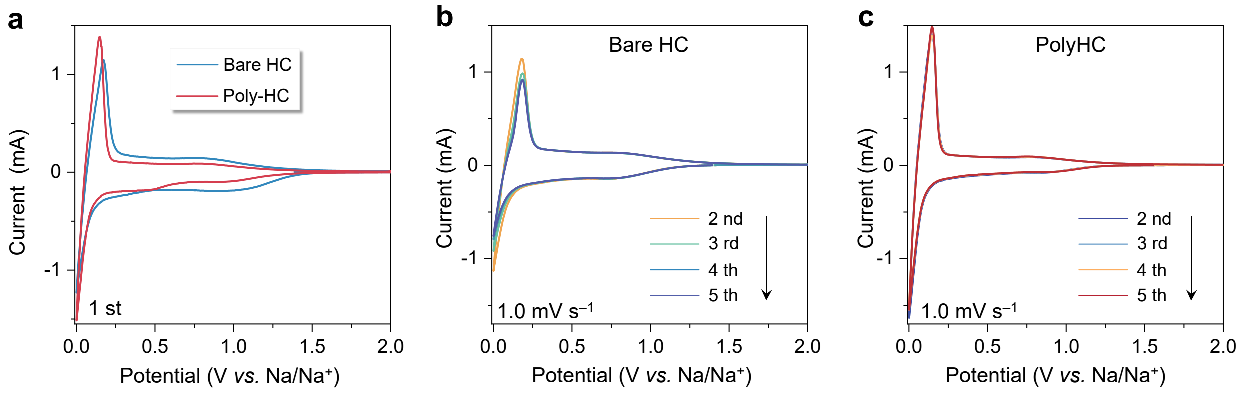

Fig. S34. (a) The initial CV curves of bare HC and PolyHC at a scanning rate of 0.1 mV s^{-1}. The CV curves of (b) bare HC and (c) PolyHC from the 2 nd to the 5 th cycles at a sweep rate of 0.1 V s^{-1}.

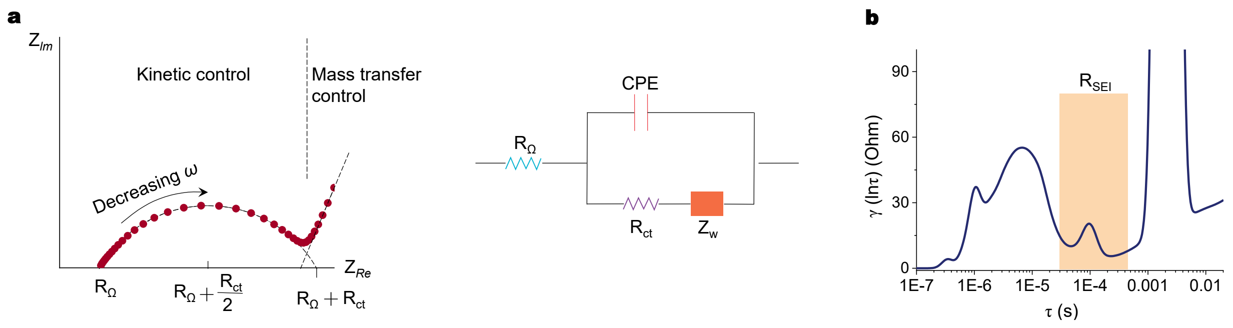

Fig. S35. (a) Selected Nyqusit curve analysis. (b) Analysis of DRT curves fitted by Nyqusit curves.

Note: To clarify the in situ impedance results, we selected a typical Nyquist curve for analysis. R_Ω represents the contact impedance between the electrolyte and the electrode. R_{ct} is the charge transfer impedance. The high-frequency region (semicircular region) is controlled by battery dynamics, while the low-frequency region is controlled by mass transfer.[8-10] Therefore, interface transfer dynamics are primarily observed in the high-frequency range. The selected DRT curve is fitted by the EIS curve to illustrate the static changes in SEI.[8-10] The yellow area is assigned to the impedance of charge passing through the SEI (R_{SEI}).

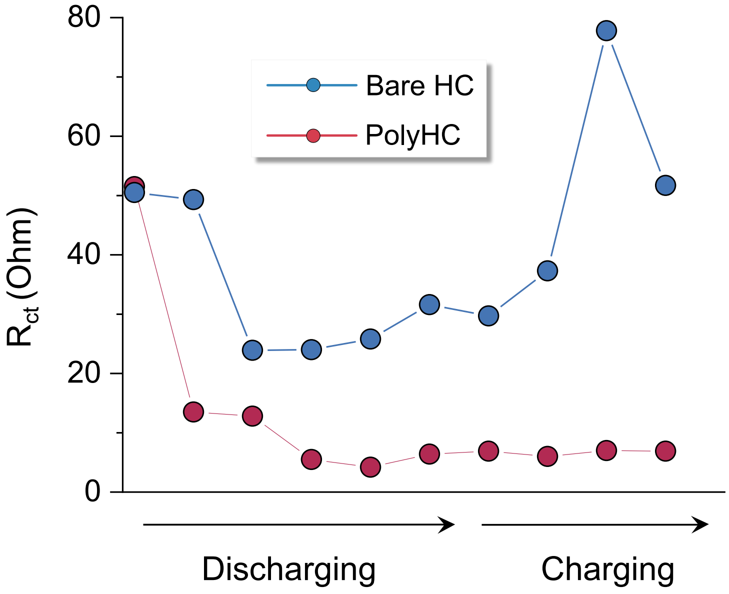

Fig. S36. The *in-situ* charge transfer impedance variation of bare HC and PolyHC during the charging and discharging process.

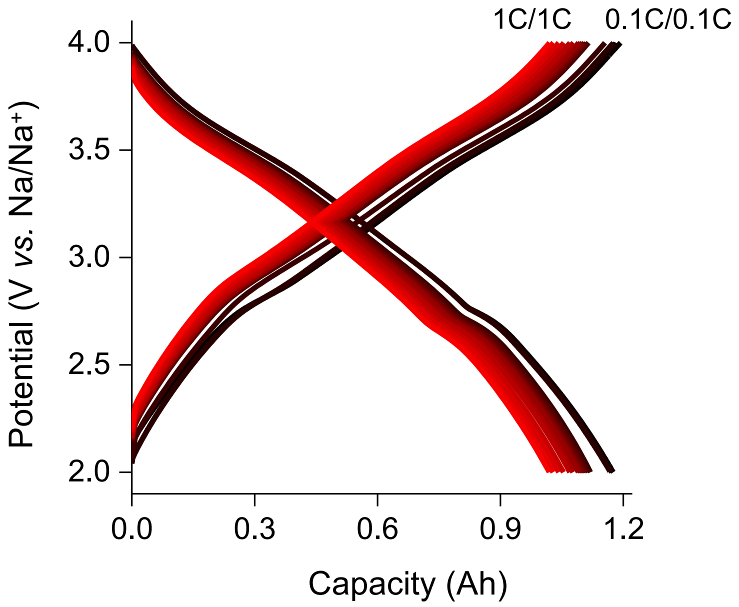

Fig. S37. The GCD profile of NFM|PolyHC SIB pouch cell.

REFERENCES

1. Izadi S, Anandakrishnan R, Onufriev A, Building water models: a different approach, *J Phys Chem Lett* 2014; **5**: 3863–71.
2. Eggimann B, Sunnarborg A, Stern H *et al.* An online parameter and property database for the TraPPE force field, *Mol Simulat* 2014; **40**: 101–5.
3. Lu T, A comprehensive electron wavefunction analysis toolbox for chemists, Multiwfn, *J Chem Phys* 2024; **161**: 082503.
4. Lu T, Chen F, Multiwfn: A multifunctional wavefunction analyzer, *J Comput Chem* 2012; **33**: 580–92.
5. Gao Y, Yan Z, Gray J *et al.* Polymer-inorganic solid-electrolyte interphase for stable lithium metal batteries under lean electrolyte conditions, *Nat Energy* 2019; **18**: 384–89.
6. Chalker J, Coddington P, Percolation, quantum tunnelling and the integer Hall effect, *J Phys C: Solid State Phys* 1988; **21**: 2665–79.
7. Cheng H, Ma Z, Kumar P *et al* High voltage electrolyte design mediated by advanced solvation chemistry toward high energy density and fast charging lithium-ion batteries, *Adv. Energy Mater* 2024; **14**: 2304321.
8. Gaberšček M, Understanding Li-based battery materials via electrochemical impedance spectroscopy, *Nat Commun* 2021; **12**: 6513–17.
9. Hu W, Peng Y, Wei Y *et al* Application of electrochemical impedance spectroscopy to degradation and aging research of lithium-ion batteries, *J Phys Chem C* 2023; **127**: 4465–95.
10. Lu Y, Zhao C-Z, Huang J-Q *et al* The timescale identification decoupling complicated kinetic processes in lithium batteries, *Joule* 2022; **6**: 1172–98.
